# Chlorosulfonic acid coated on porous organic polymer as a bifunctional catalyst for the one-pot three-component synthesis of 1,8-naphthyridines†

**DOI:** 10.1039/d2ra05070f

**Published:** 2022-09-28

**Authors:** Ramin Ghiai, Sedigheh Alavinia, Ramin Ghorbani-Vaghei

**Affiliations:** Department of Organic Chemistry, Faculty of Chemistry, Bu-Ali Sina University Hamedan 6517838683 Iran rgvaghei@yahoo.com ghorbani@basu.ac.ir +98(81)38380647

## Abstract

The synthesis of six-membered oxygen- and nitrogen-containing heterocycles has been regarded as the most fundamental issue in organic chemistry and the chemical industry because these heterocycles are used in producing high-value products. In this study, an efficient, economic, sustainable, and green protocol for their multicomponent synthesis has been developed. The one-pot direct Knoevenagel condensation–Michael addition–cyclization sequences for the transformation of aromatic aldehydes, malononitrile, and 2-aminopyridine generate the corresponding 1,8-naphthyridines over a novel mesoporous bifunctional organocatalyst supported cholorosulfonic acid [poly(triazine-benzene sulfonamide)*-*SO_3_H (PTBSA-SO_3_H)] under ambient conditions. The catalyst was used for the formation of 1,8-naphthyridine derivatives for six runs. The current strategy provided a wider substrate range, and short reaction times.

## Abstract 2

Chlorosulfonic acid coated on porous organic polymer as a bifunctional catalyst for one-pot three-component synthesis of 1,8-naphthyridines

## Graphical abstract

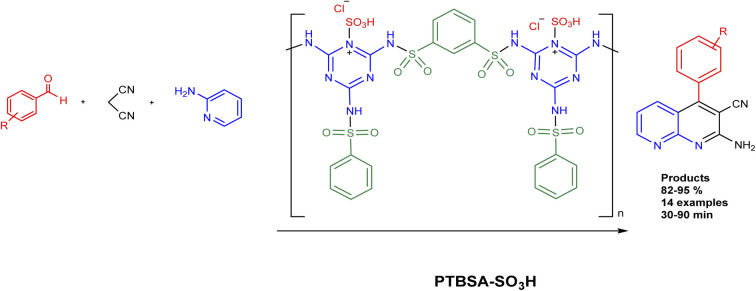

## 1. Introduction

Nowadays, the scientific community is continuously striving toward the refinement of materials that lead to sustainable development.^1,2^ The development of integrated processes that produce chemicals and biological materials in a safe and affordable way is one of today's controversial topics that has attracted particular attention in a variety of studies.^3,4^ In recent years, the development of heterogenization of homogeneous catalysts and use of these materials which support the fundamental pillars of green chemistry is a demanding task in chemistry.^5–8^ Recently, the synthesis of solid acid catalysts used as heterogeneous catalysts has attracted attention in various organic reactions.^3,4,9–11^ Today, there is increasing interest in the design and synthesis of heterogeneous supports containing high loads of sulfonic acid groups to increase the turnover number (TON) and turnover frequency (TOF) of the reaction.^12^ Despite significant advances in producing eco-friendly heterogeneous supports (such as cellulose,^13^ silica,^14,15^ carbon nanotubes,^16^ iron aluminate spinel hercynite,^17^ graphite carbon nitride,^18^ magnetic nanoparticles,^4,5,7,19–21^ and polymers^22^), it is necessary to set certain rules for their applications in terms of their synthesis, recycling, management, and cost.

In recent years, the development of porous organic polymers (POPs) as heterogeneous supports for the immobilization of SO_3_H groups for various chemical transformations has received considerable attention as a new strategy to address the energy and sustainability challenges. However, many POP synthesis routes require precious metal catalysts, which are typically not recycled, significantly driving up cost and hindering scale-up. Furthermore, specifically polymerisable groups in monomeric material are often required for the formation of POP networks. Such monomers are seldomly commercially available or are expensive. In this regard, cross-linked polysulfonamides have emerged as an innovative field due to their high activity, selectivity, chemical inactivity, and excellent thermal stability.^23–26^ Continuing from previous work on the preparation of porous/crosslinked polysulfonamides,^26–32^ this study incorporates a hydrophilic triazine linker into the hydrophobic aromatic skeleton of the polysulfonamide and immobilizes chlorosulfonic acid on the prepared support (PTBSA).

Poly(triazine-benzene sulfonamide) is one of the non-metal substrate, being nontoxic with high chemical and thermal stability, resistant to acidic and basic conditions and various solvents. These unique properties are due to the presence of nitrogen atoms in the carbon architecture of prepared support (PTBSA), which can be prepared easily from cheap nitrogen -rich precursors such as melamine. Because of these great features, nowadays, polysulfonamides is widely applied in different studies such as drug synthesis,^33^ and heterogeneous catalyst.^20^ In addition, PTBSA provide bifunctionality in the reaction mixture in the form of acid/base sites. Therefore, its use as a catalyst in organic synthesis could be beneficial as the development of new methods for the synthesis of heterocyclic compounds plays a significant role in organic syntheses.

**Scheme 1:**
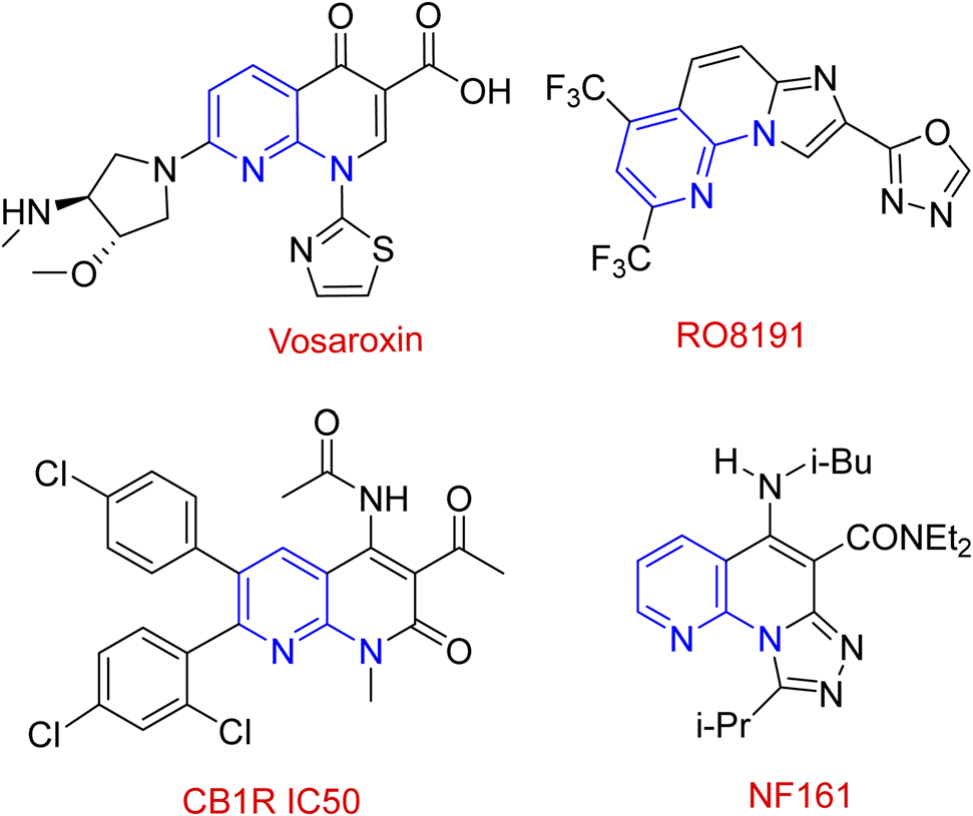
Some drugs containing naphthyridine structure.

Domino multicomponent reaction (MCR) inevitably engrossed the organic chemists as it not only encompasses the merits of multicomponent reactions but also addresses its demerits such as more wastage and lower yield.^34^ It makes the process benign by curtailing the amount of solvent, cost and energy during the reaction.^35^ Among various discovered and synthesized heterocyclic systems, the fused nitrogen-containing heterocycles are ubiquitous. 1,8-Naphthyridines is one of the privileged pharmaceutical and pharmacologically active ingredients that has received considerable interest from both synthetic and industrial perspectives.^36–38^ A variety of 1,8-naphthyridines derivatives, namely jasmine, perolidine, and sublin, are used directly as antibacterial, antiviral, anti-inflammatory, antitumor, and antioxidant (Scheme 1).^39–42^ Despite the synthetic methods described for syntheses of these materials,^43–45^ we believe there is still work to do in this field. Hence, it is necessary to develop additional green, atom-efficient, sustainable, and high-yielding methods for synthesizing these compounds.

Herein, regarding the pursuing our interest in porous substances as catalysts in MCRs,^17,46,47^ we aimed to design and prepare a stable multifunctional crosslinked polymer (PTBSA-SO_3_H) consisting of a melamine, sulfonamide and sulfonic acid. Accordingly, it was tried to develop a very efficient heterogeneous catalyst to synthesize 1,8-naphthyridines under ambient conditions.

## 2. Experimental

### 2.1. Materials and instruments

Aldehydes, triazine, benzene-1,3-disulfonyl chloride, 2-aminopyridine, malononitrile, chlorosulfonic acid, and solvents were purchased from Sigma-Aldrich Company. Also, the thin-layer chromatography (TLC) of the commercial plates (silica gel 60 F254), were purchased from Merck Company. FT-IR spectra were recorded in a spectrophotometer (PerkinElmer 781). To investigate the surface morphology of the catalyst FE-SEM images and EDX analyses provided by a Sigma ZEISS, Oxford Instruments Field Emission Scanning Electron Microscope. The morphology of prepared catalysts was investigated using TEM by a Philips CM 120, Netherlands and microscope with an accelerating voltage of 150 kV. X-ray diffraction was performed using a Philips X'pert MPD diffractometer with a Cu operating at a current of 100 mA and a voltage of 45 kV, with the Cu-Kα radiation (*λ* = 0.154056 nm) at the 2q range of 10–80 and scanning at the speed of 0.05° per minute. Thermogravimetric analysis (TGA) was carried out using Shimadzu DTG-60 instrument at 25 to 600 °C. The pore volume and pore size distribution were resulted from the desorption profiles of the isotherms using the Barrett–Joyner–Halenda (BJH) method. NMR spectra (Bruker 400 MHz) were used to confirm product structure by DMSO-d6 as a solvent on a Bruker DRX-400 spectrometer.

### 2.2. Synthesis of poly(triazine-benzene sulfonamide)*-*SO_3_H (PTBSA-SO_3_H)

**Scheme 2:**
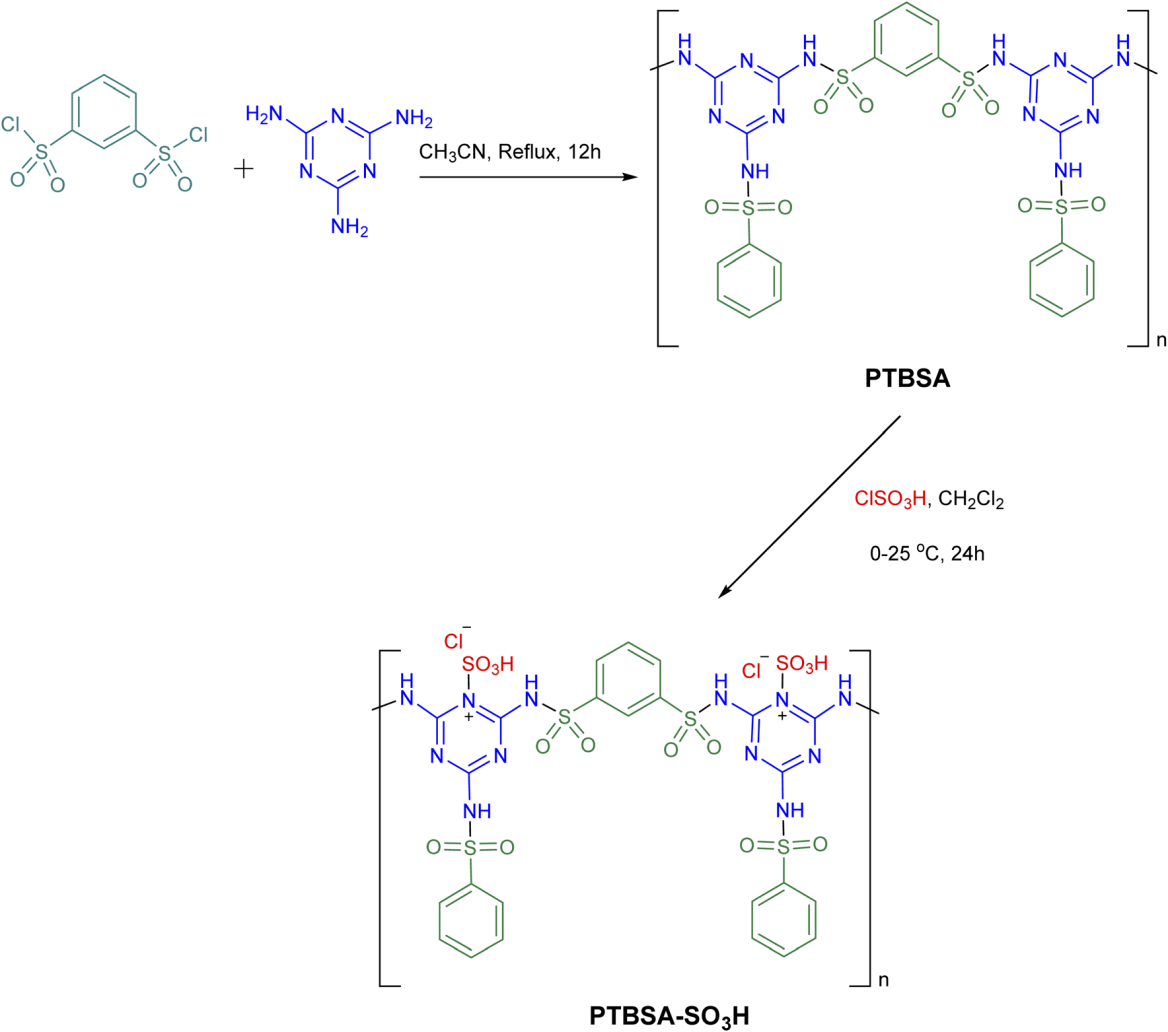
The general route for the synthesis of PTBSA-SO_3_H.

In the first step, the poly(triazine-benzene sulfonamide) (PTBSA): was prepared according to the our previous typical procedure:^5^ for this purpose, in a round-bottom flask, the prepared benzene-1,3-disulfonyl chloride (1 mmol) and CH_3_CN (10 mL) was placed and stirred for 10 min. Thereafter, triazine (0.7 mmol) was put in the above-mixed system under continuous stirring at reflux condition for 12 h. Then, the reaction mixture cooled to room temperature and PTBSA was collected by centrifugation. The came into possession of PTBSA was rinsed three times with acetonitrile, and dried under vacuum at 80 °C for 24 h. Afterward, PTBSA (1 g) was dispersed in dichloromethane (25 mL) in a 250 mL round button flask, to which we added chlorosulfonic acid (10 mmol in 10 mL CH_2_Cl_2_). After reacting for 24 hours, the solid was filtered and washed with deionized water and EtOH, in the order of their appearance, and the residues were dried at 50 °C. Scheme 2 illustrates the preparation route of this residue.

### 2.3. Preparation of 1,8-naphthyridine derivatives using PTBSA-SO_3_H

**Scheme 3:**
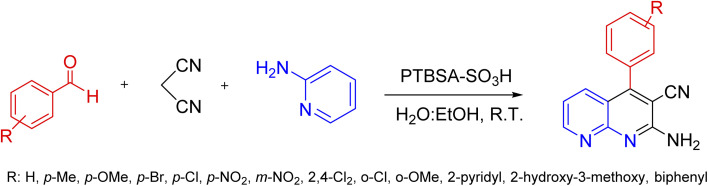
Synthesis of 1,8-naphthyridine derivatives using PTBSA-SO_3_H.

A mix of 2-aminopyridine (1 mmol), malononitrile (1 mmol), aldehyde (1.2 mmol), and H_2_O : EtOH (1 : 1, 2 mL) was put in a 10 mL round-bottomed flask in the presence of PTBSA-SO_3_H catalyst (0.08 g) and shaken vigorously at room temperature. After completing the reactions by TLC (10 : 3, *n*-hexan : ethylacetate), the catalyst was gathered by centrifugation. Next, it was recovered by washing with H_2_O/EtOH. The precipitated product was collected and recrystallized in hot EtOH. Finally, the obtained product and PTBSA-SO_3_H was dried at 50–60 °C for 5 h (Scheme 3). The products were characterized based on their physical and spectral data obtained from electrospray ionization (ESI).

## 3. Result and discussion

### 3.1. Catalyst characterization

The resulted PTBSA and PTBSA-SO_3_H was characterized by various analytical techniques, including Fourier transform infrared spectroscopy (FT-IR), X-ray diffraction (XRD), scanning electron microscopy (SEM), TEM, energy-dispersive X-ray spectroscopy (EDS), nitrogen absorption-desorption experiment (BET), and thermal gravimetric analysis.

#### 3.1.1. FT-IR studies

**Fig. 1:**
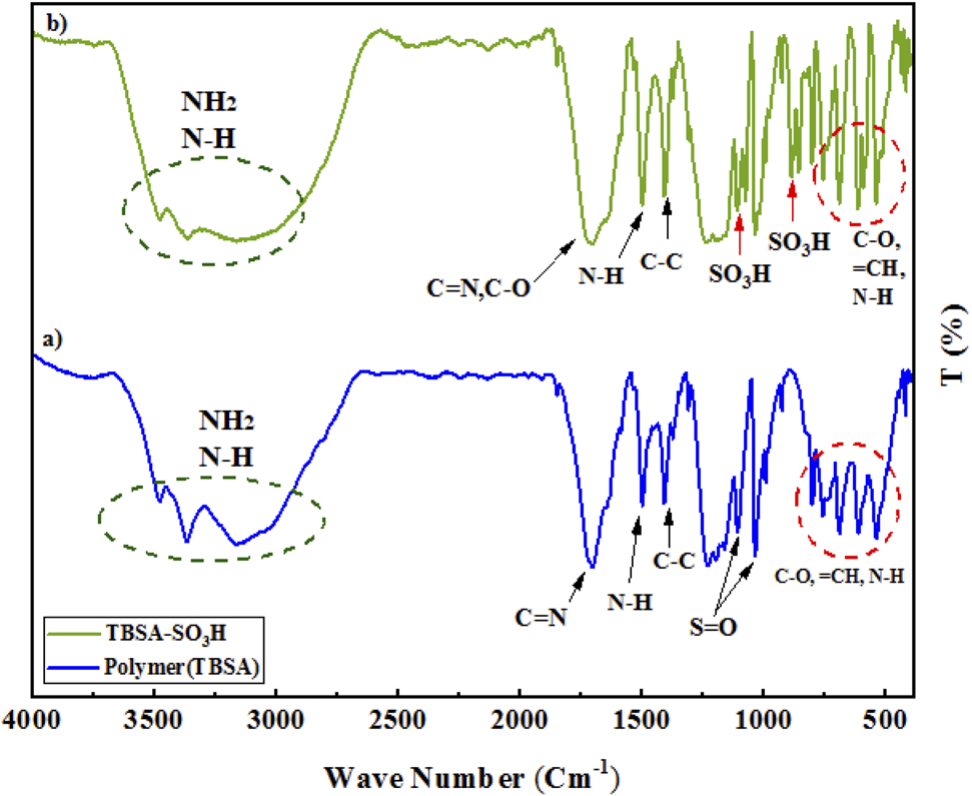
FT-IR spectra of porous PTBSA (A), PTBSA-SO_3_H (B).

In Fig. 1, the FT-IR spectra of PTBSA (Fig. 1A) and PTBSA-SO_3_H (Fig. 1B) over the range of 500–4000 cm^−1^ are shown. In FT-IR spectrum of PTBSA (Fig. 1A) the broad band at 3200–3700 cm^−1^ is indicated the presence of numerous N–H groups in PTBSA. Also, the absorption bands at 1681 cm^−1^, and 1630 cm^−1^ correspond to C=N groups, indicating the characteristic absorption peaks of the triazine groups. The absorption at 1115 cm^−1^, and 1339 cm^−1^ indicate the formation of sulfonamide bands. In the PTBSA-SO_3_H spectrum, all peaks in spectrum (a) are observed. Further, the appearance of intense band at 854, 1075 and 1208 cm^−1^ assigned to SO_3_H vibration frequency, which confirms the immobilization of chlorosulfonic acid over PTBSA support (Fig. 3B).^7^ The difference between PTBSA and PTBSA-SO_3_H were verified the scaffold of the catalyst.

#### 3.1.2. FE-SEM and TEM studies

**Fig. 2:**
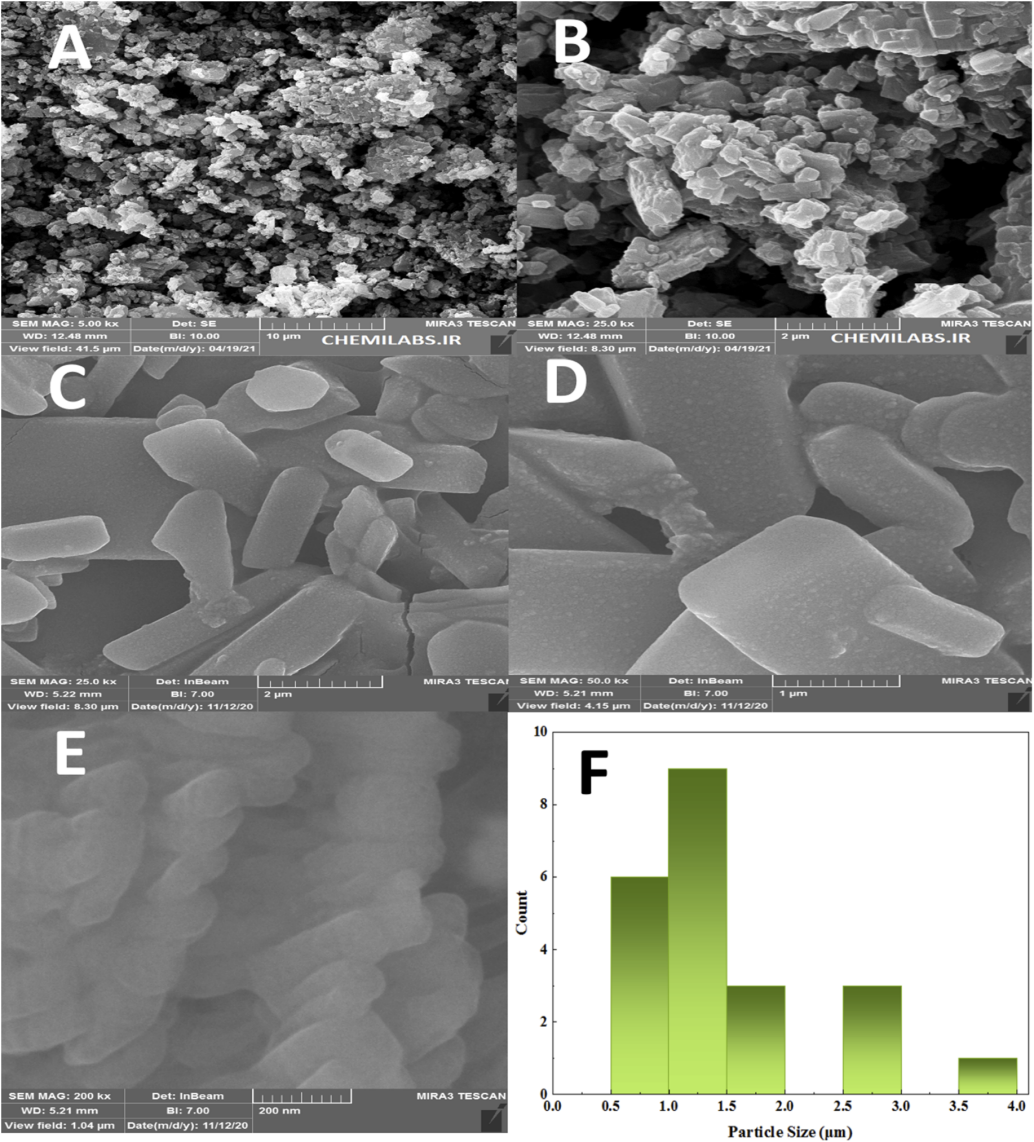
FE-SEM photographs of PTBSA (a) and (b), PTBSA-SO_3_H (c) and (e) and particle size distribution of PTBSA-SO_3_H (f).

**Fig. 3:**
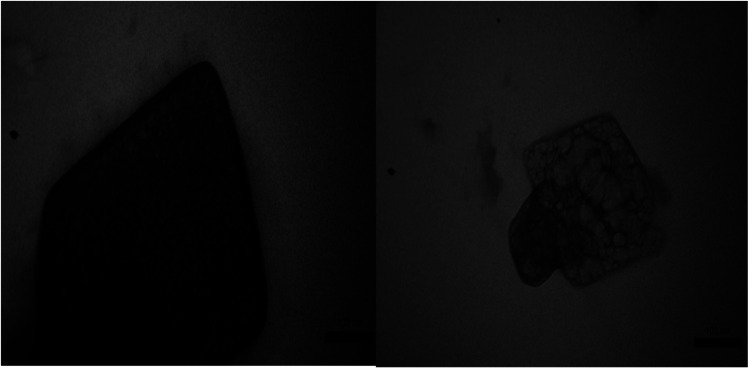
TEM photographs of PTBSA-SO_3_H.

The SEM technique was used to study the morphology, crystal structure and size distribution of the synthesized PTBSA and PTBSA-SO_3_H catalyst. In this figure, the FE-SEM photos of PTBSA (Fig. 2A and B) and PTBSA-SO_3_H (Fig. 2C and E) have layered shapes. Moreover, surface modification has not considerably changed the morphology of the PTBSA. In addition, the average size of the nickel ferrite nanoparticles was also determined through the histogram shown in the inset Fig. 2F, the particle size is in the range of 0.5 to 4 μm. The uniform size are critical parameters in catalytic activity. TEM micrographs were analyzed for comprehending the morphology, particle size and distribution of layered of PTBSA. TEM images of PTBSA-SO_3_H show the mesoporous structure with no aggregation (Fig. 3).

#### 3.1.3. Elemental composition studies

**Fig. 4:**
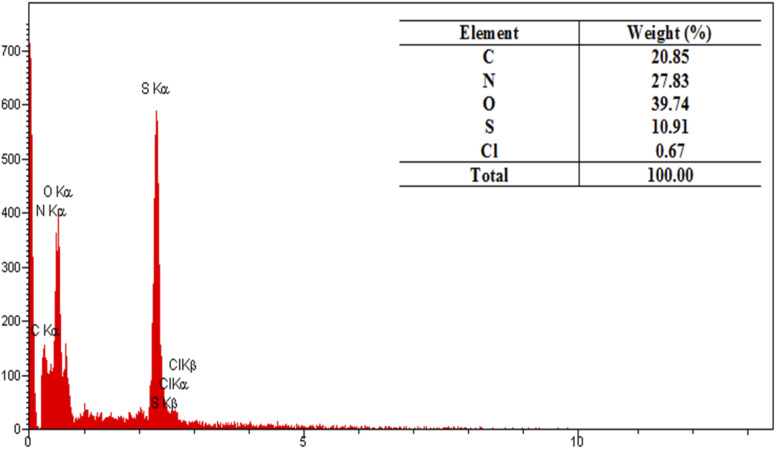
EDX spectrum of PTBSA-SO_3_H.

**Fig. 5:**
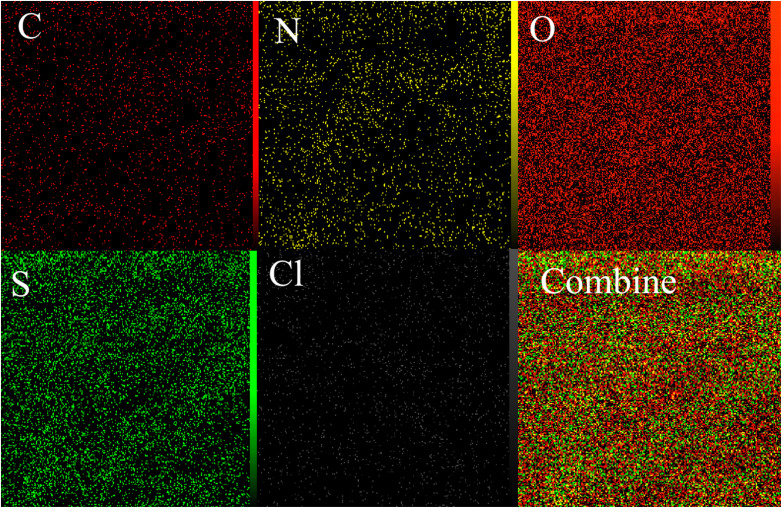
Elemental mapping of the C, N, O, S and Cl atoms achieved from SEM micrographs.

In this study, the EDX analysis (Fig. 4) was conducted to study the elemental composition of the PTBSA-SO_3_H adsorbents. This method confirms the presence of all studied elements in these adsorbents. In addition, the elemental mapping in Fig. 5 indicates that these elements are unformed distributed. Furthermore, the presence of chlorosulfonic acid immobilized on the PTBSA surface is attributed to chlorine molecules detected in the elemental analysis (Fig. 4 and 5).

#### 3.1.4. Porosity studies

**Fig. 6:**
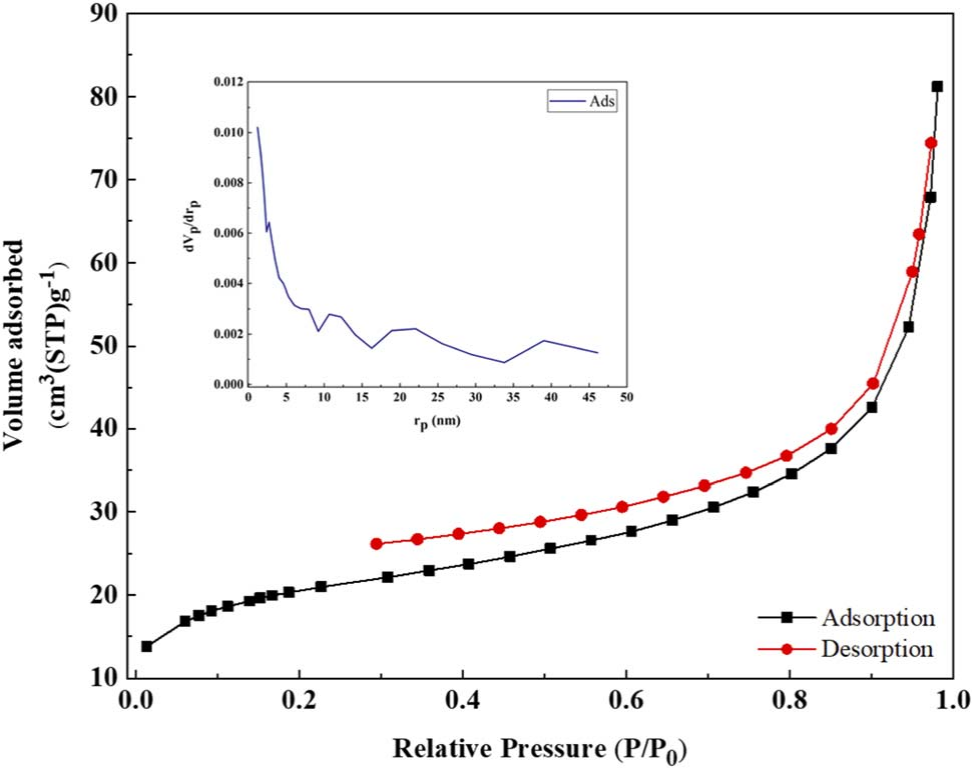
N_2_ adsorption–desorption isotherms for PTBSA.

The pore property of the PTBSA was measured by Nitrogen sorption (Fig. 6). The results showed a type-IV isotherm (because of the mesoporous materials) and a type-H3 hysteresis loops (defined by IUPAC) (Fig. 6). Table 1 represents the BET experimental results for PTBSA system.^47^ According to the BJH method measurements, the cumulative adsorption surface area of the nanocomposite is 75.46 m^2^ g^−1^ and the pore diameter is 1.64 nm.

**Table 1:** Results of the Langmuir and BET measurements

Parameter	PTBSA
*a*_s_ (m^2^ g^−1^)	75.46
*V*_m_ (cm^3^(STP)/g)	20.58
*V*_p_ (cm^3^ g^−1^)	0.10
*r*_p_ (nm)	1.64
*a*_p_ (m^2^ g^−1^)	25.03

#### 3.1.5. Thermogravimetric analysis

**Fig. 7:**
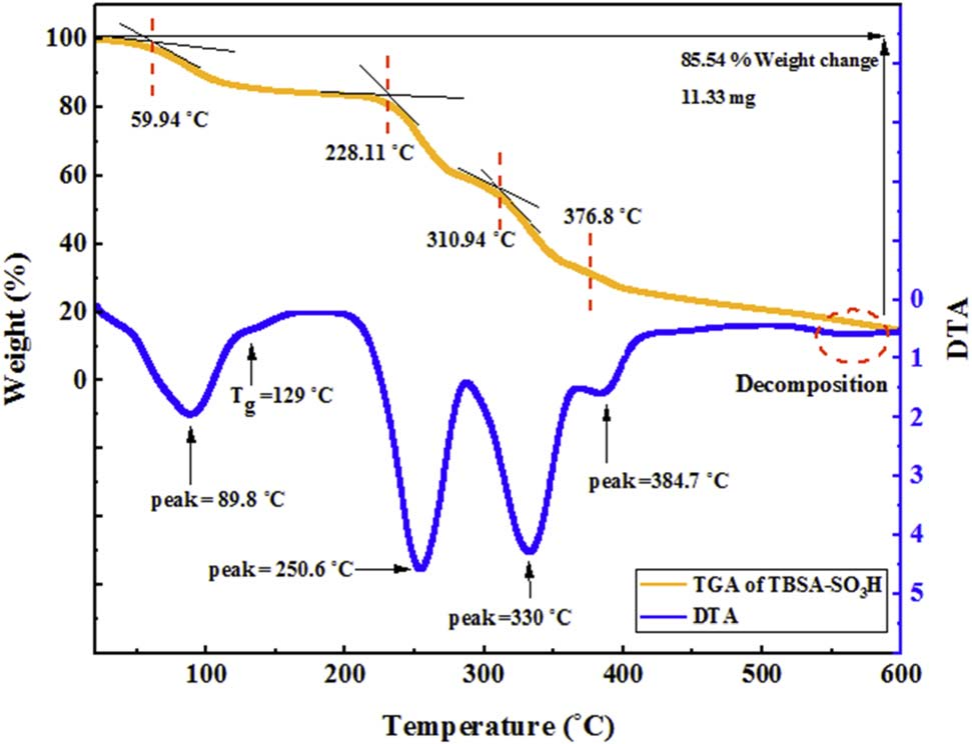
TGA curve of PTBSA-SO_3_H.

The thermogravimetric analysis (TGA) was used to study of stability and the weight losing of the synthesized catalyst (Fig. 7). As observed from this curve, losing 1.7% of the weight between 40–150 °C could be related to loss of moisture and solvents. Also, weight losing in the temperature range of 150–600 °C indicates removing of organic moieties such as triazine, 1,3-benzene-disulfonylchloride, and decomposition of the composite. The TGA curve of PTBSA-SO_3_H shows a five-step degradation, in which 85.54% of the sample was lost at 100 °C, 129 °C, 250.6 °C, 330 °C and 384 °C respectively. In addition, the DTA diagrams indicate that glass transition point (*T*_g_) is 129 °C.

#### 3.1.6. Titration for the determination of SO_3_H group density

The total acidity of the PTBSA-SO_3_H catalyst was found to be 8 mmol g^−1^ through back titration method.

#### 3.1.7. Investigation of amphiphilicity of PTBSA-SO_3_H

**Fig. 8:**
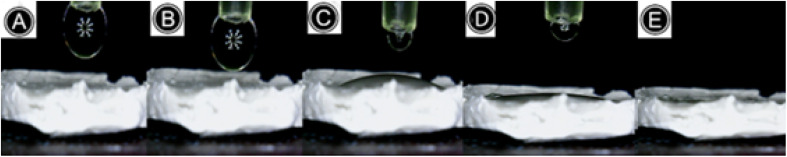
Contact angles of PTBSA-SO_3_H: photograph of water droplets.

**Fig. 9:**
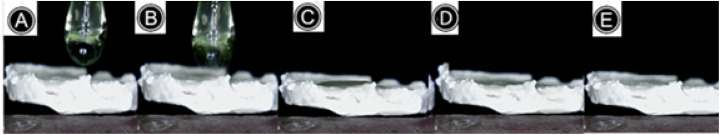
Contact angles of PTBSA-SO_3_H: photograph of oil droplets.

The amphiphilicity of the prepared adsorbent was investigated by performing the contact angle (CA) measurements. Fig. 8 and 9 show the CAs of PTBSA-SO_3_H for both oil and water droplets. As can be seen from Fig. 8D, the CA estimated between water and PTBSA-SO_3_H (0°) indicates the hydrophilicity of the prepared adsorbent. Moreover, this figure shows the quick adsorption of the oil droplet and the CA of about 0° (Fig. 9D).

### 3.2. Catalytic studies

#### 3.2.1. Effect of reaction parameters

After design, synthesis and characterization of PTBSA-SO_3_H, application of it in the synthesis of 1,8-naphthyridines derivatives was studied. To this end, 2-amino-4-phenyl-1,8-naphthyridine-3-carbonitrile was prepared *via* the reaction of 2-aminopyridine, benzaldehyde, and malononitrile with various amounts of PTBSA-SO_3_H (Table 2). The results showed a low yield (*i.e.*, 10%) of PTBSA-SO_3_H after 24 h (entry 1) in the absence of the catalyst. Studying various amounts of PTBSA-SO_3_H (entries 2–4) showed that 0.08 g (entry 3) yields the most desired catalytic outcomes. However, exceeding the catalyst content by 0.1 g does not affect the reaction time and yield (entry 4). In the next step, the temperature effect on the process was examined by performing the model reaction at 50 °C (entry 5). Based on the obtained results, exceeding the room temperature does not influence reaction time and yield. Then, the reaction under consideration has been screened in the presence of variety of solvent systems such as EtOH : H_2_O, EtOH, toluene, THF, CH_3_CN, DMF, H_2_O, and solvent-free condition (entries 6–12). It was noted that best results were obtained in EtOH : H_2_O (1 : 1). Finally, comparing the catalysts' performance using melamine, and PTBSA revealed the failure of the reaction (entries 13–14).

**Table 2:** Optimization of reaction conditions

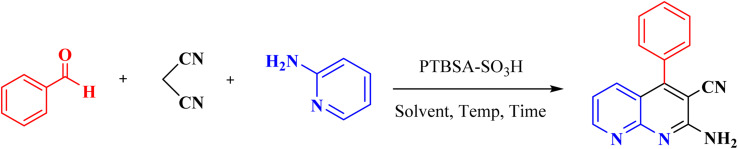					
Entry	Cat. (g)	Solvent	Temperature (°C)	Time (h)	Yield^a^ (%)
1	—	EtOH : H_2_O	R.T.	24	10
2	0.05	EtOH : H_2_O	R.T.	1	88
3	0.08	EtOH : H_2_O	R.T.	0.5	95
4	0.1	EtOH : H_2_O	R.T.	0.5	96
5	0.08	EtOH : H_2_O	50 °C	0.5	95
6	0.08	Solvent-free	R.T.	2	65
7	0.08	EtOH	R.T.	1	85
8	0.08	DMF	R.T.	1	80
9	0.08	Toluene	R.T.	2	80
10	0.08	CH_3_CN	R.T.	2	70
11	0.08	THF	R.T.	1	78
12	0.08	H_2_O	R.T.	2	50
13	0.08^b^	EtOH : H_2_O	R.T.	1	90
14	0.08^c^	EtOH : H_2_O	R.T.	1	42

a Isolated yield.

b The model reaction was examined in the presence of PTBSA.

c The model reaction was examined in the presence of melamine.

#### 3.2.2. Effect of different aldehydes on the catalytic activity of PTBSA-SO_3_H

This method's efficiency was confirmed by the employment of the one-pot three component reaction of 2-aminopyridine, malononitrile and derivatives of aldehydes. In this way, we could obtain the desired 1,8-naphthyridines under the optimized reaction conditions (Table 3). Overall, the results did not show any significant effect of these material groups on the yields. Eventually, the desired catalysts were obtained in excellent yields of 85–95%. Besides, electron-donating aldehydes declined the reaction rate (entries 7–10). Generally, this method indicated a high selectivity toward the products and produced no considerable byproducts. In order to investigate the efficiency of this method, different types of aliphatic aldehydes such butyraldehyde, 2-methyl butyraldehyde, hexanaldehyde, phenylpropionaldehyde and cinnamaldehyde were employed to synthesize diverse structurally functionalized of 1,8-naphthyridines derivatives, which the reaction was not successful. In aromatic benzaldehyde derivatives, the carbonyl group effectively takes part in the resonance. So, the aromatic ring on aldehyde can be stabilize the positive charge on carbonyl groups in the transition state. Therefore, the yields for aromatic benzaldehyde derivatives are higher than aliphatic compounds, probably due to the resonance effect.

**Table 3:** Synthesis of 1,8-naphthyridines using PTBSA-SO_3_H^a^

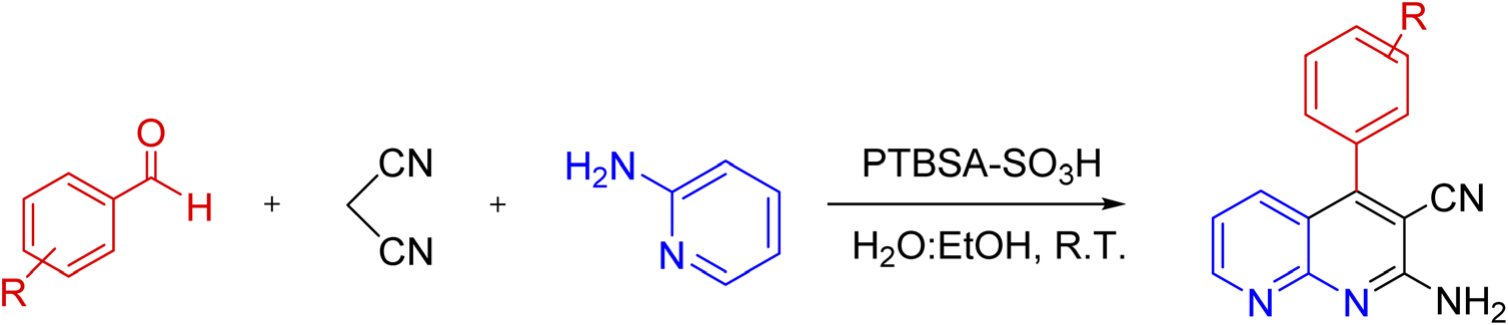						
Entry	Substrate	Product	Time (min)	Yield^b^ (%)	Melting point	
					Measured	Literature
1	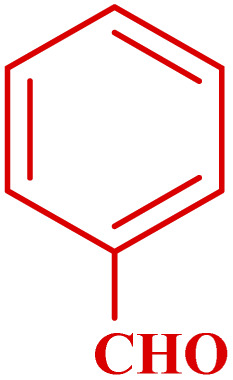	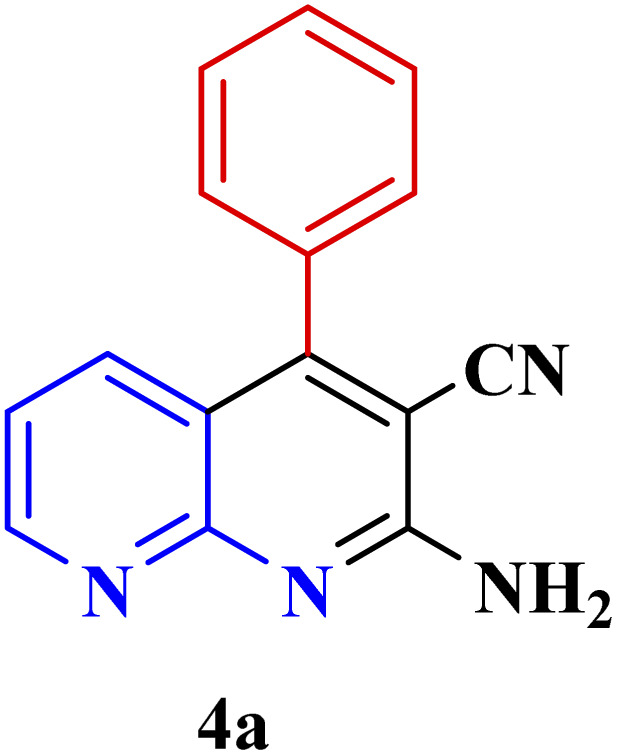	30	95	150–152	150–152 (ref. 48)
2	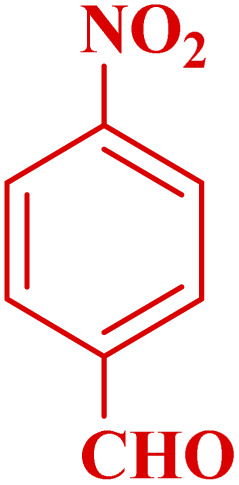	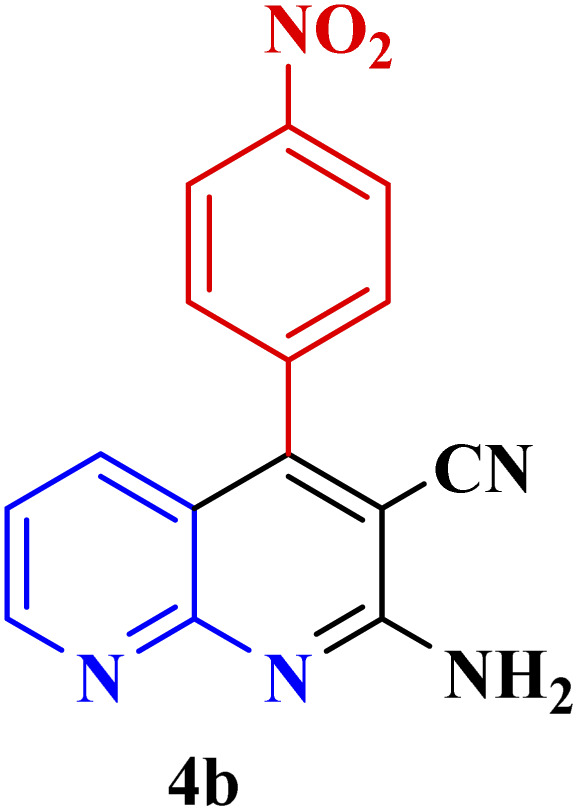	45	95	158–159	159–161 (ref. 49)
3	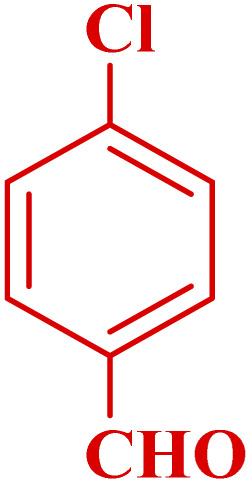	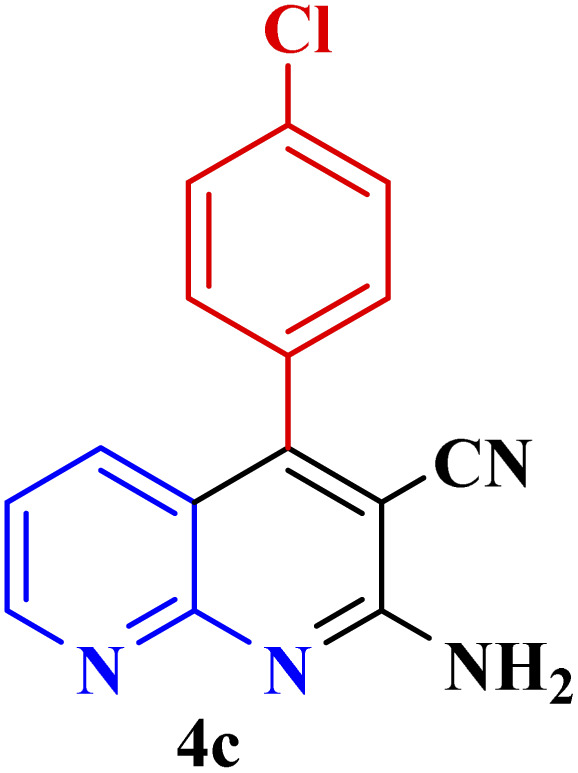	40	94	210–212	210–212 (ref. 49)
4	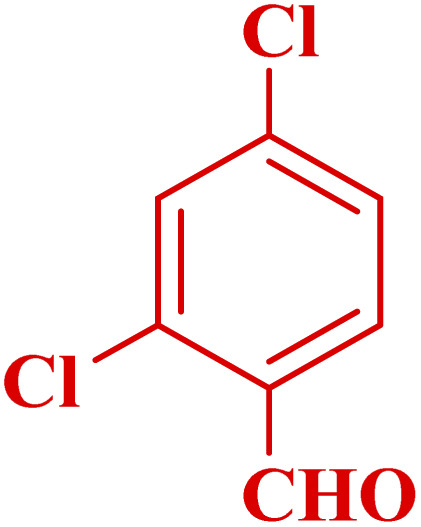	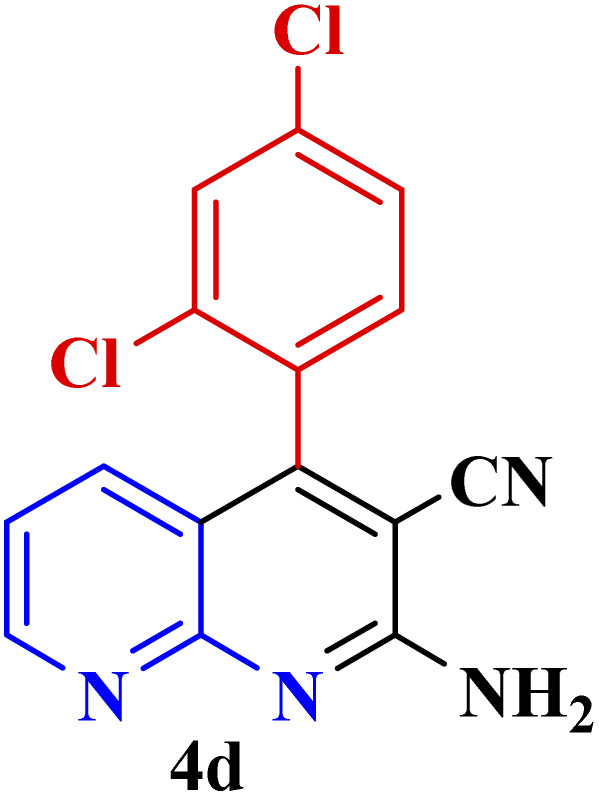	45	93	163–165 °C	164–165 (ref. 49)
5	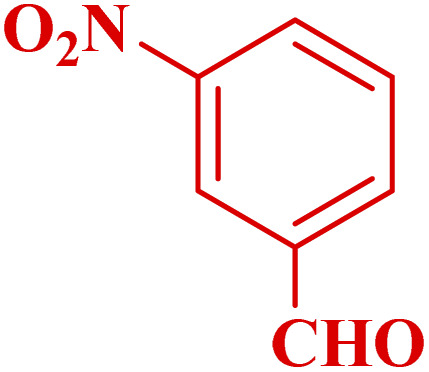	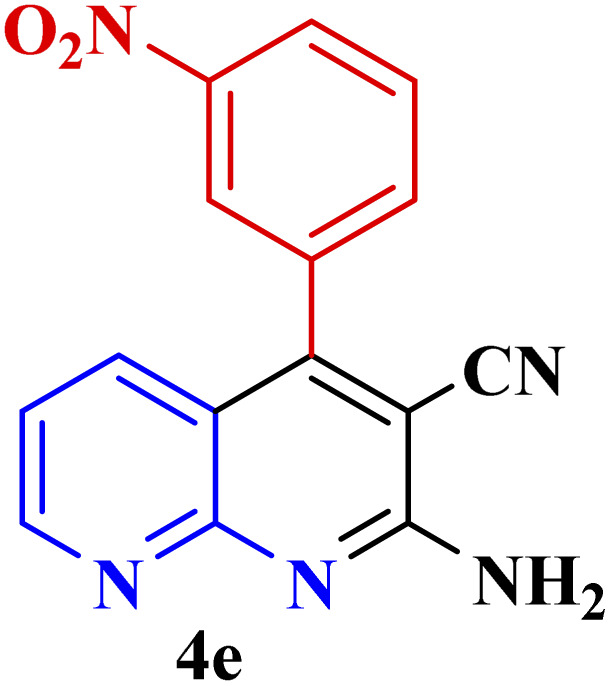	30	92	160–162 °C	160–163 (ref. 49)
6	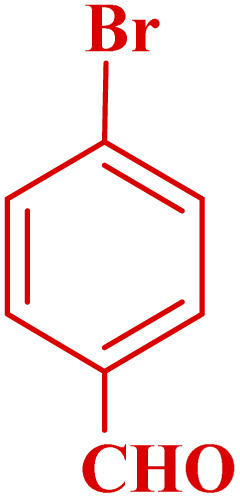	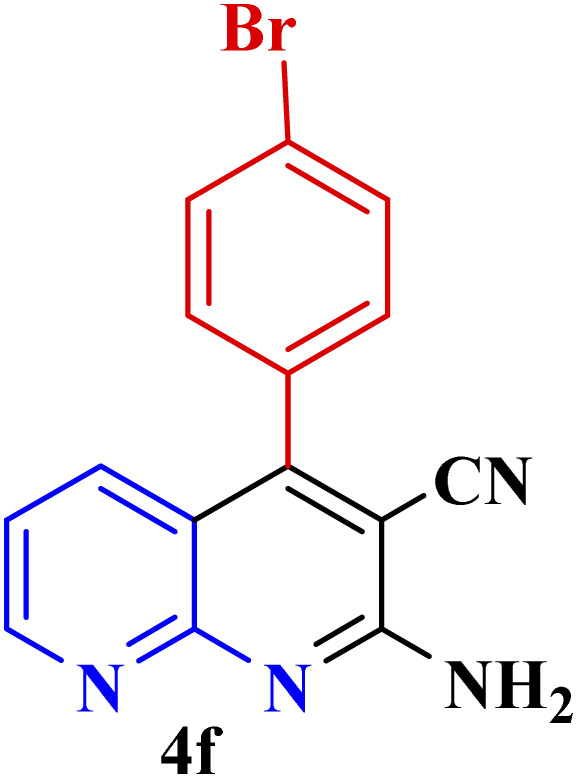	50	91	181–182	New
6	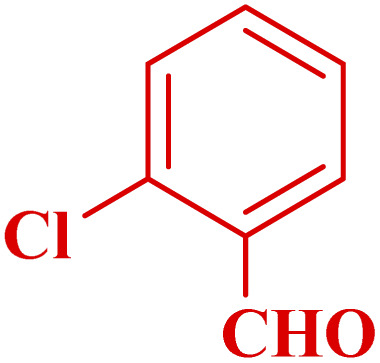	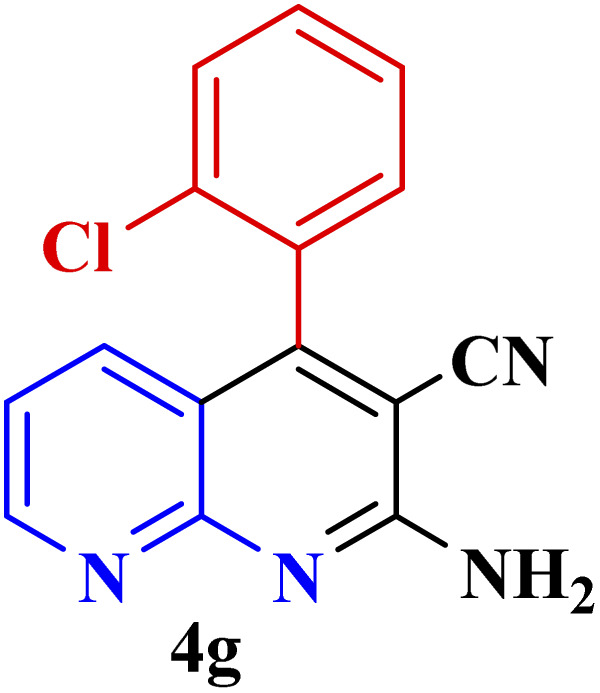	60	90	169–170	168–170 (ref. 49)
7	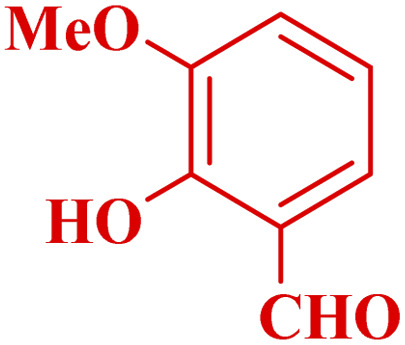	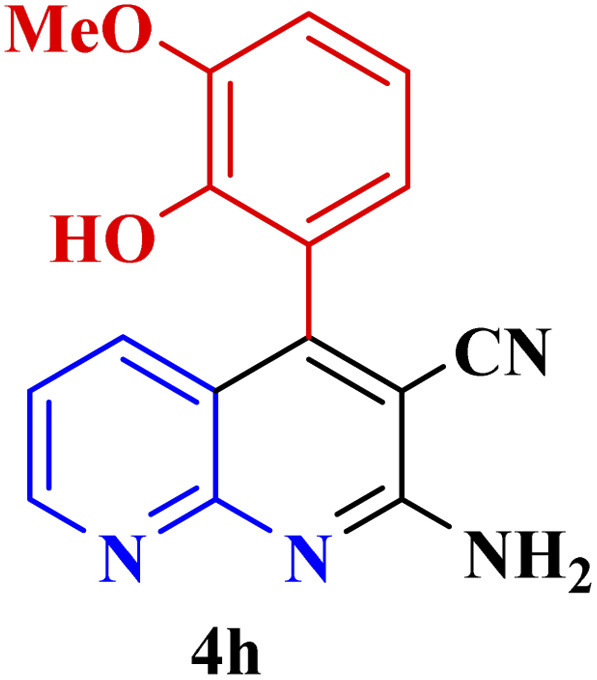	90	88	180–181	180–181 (ref. 48)
8	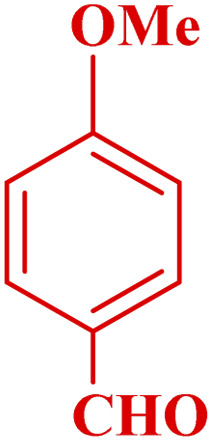	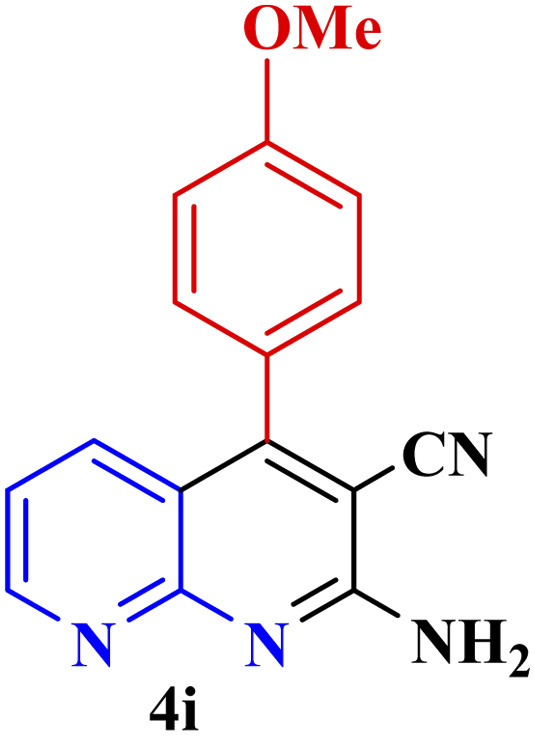	70	96	158–160 °C	157–158 (ref. 49)
9	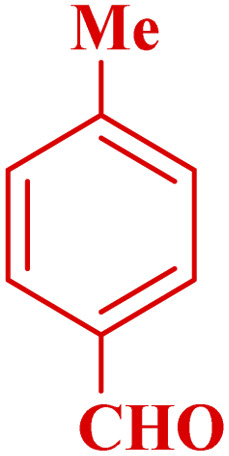	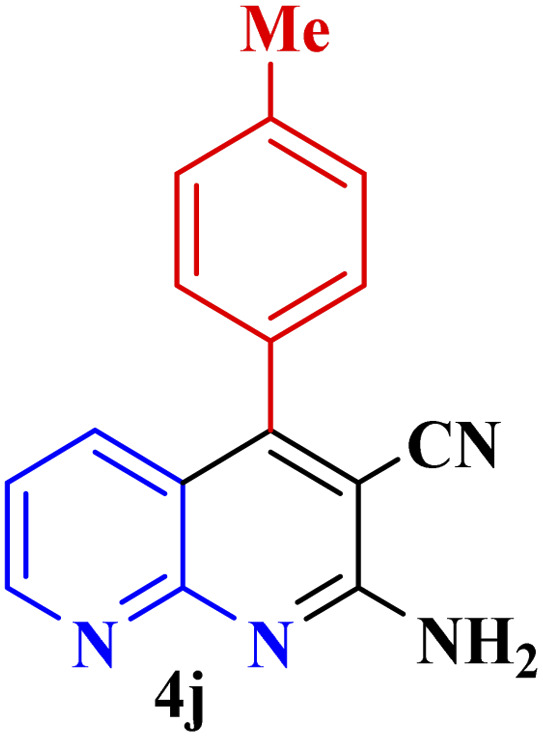	70	94	156–158	155–156 (ref. 49)
10	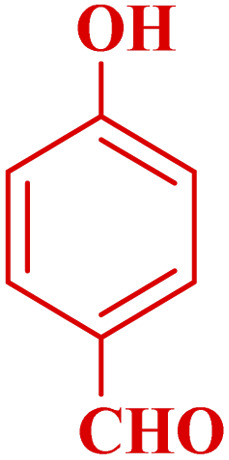	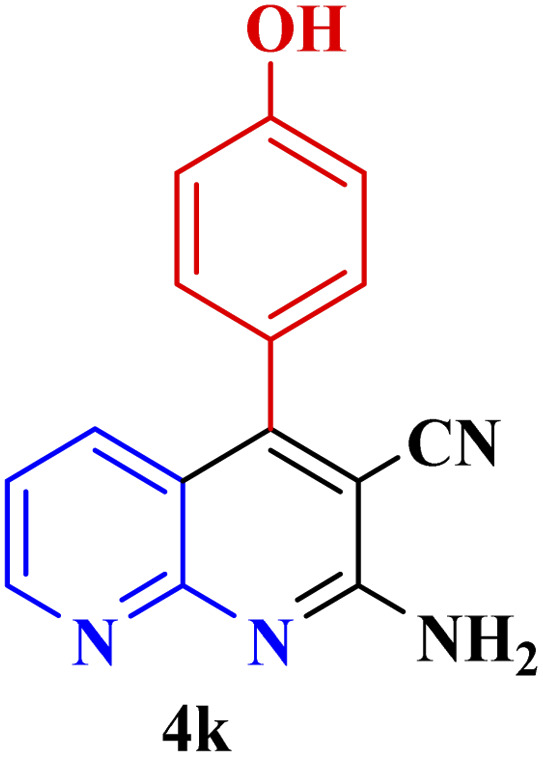	70	89	144–145	New
11	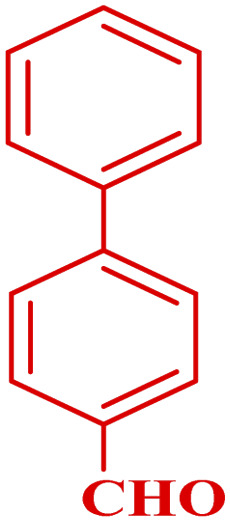	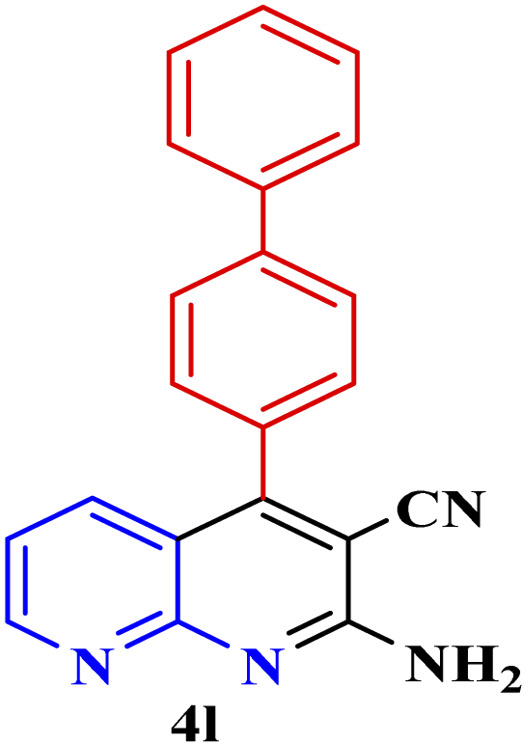	90	82	142–143	142–143 (ref. 48)
12	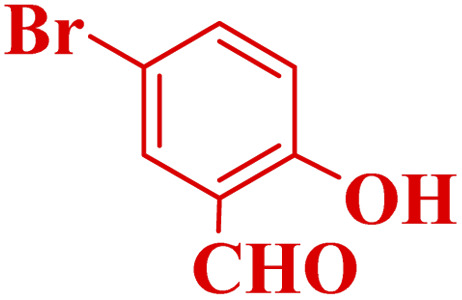	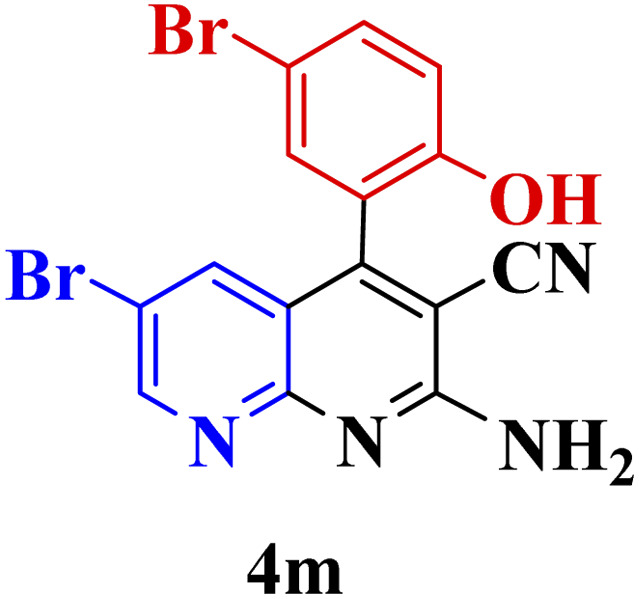	60	82	161–163	160–162 (ref. 48)
13	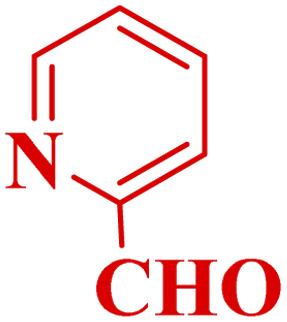	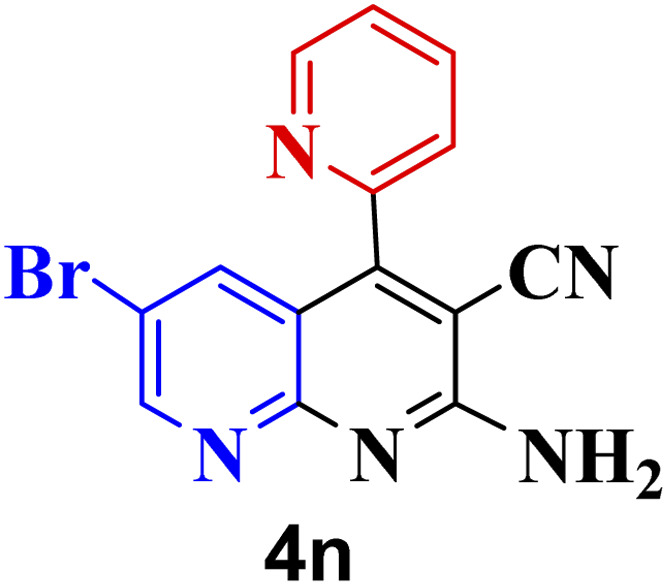	30	88	Mp > 300 °C	Mp > 300 (ref. 48)
14	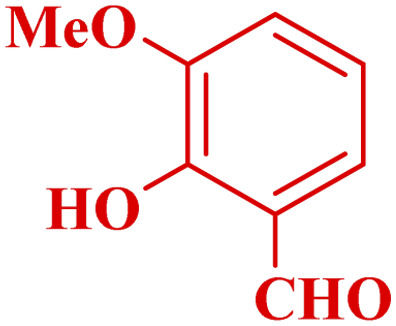	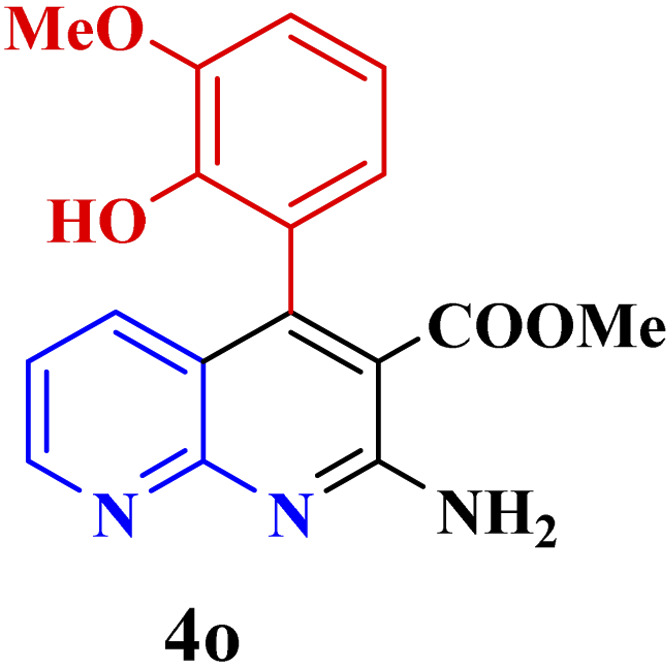	60	85	164–166	164–166 (ref. 48)

a Reaction condition: benzaldehyde derivatives (1.2 mmol), 2-aminopyridine (1 mmol), malononitrile (1 mmol), PSTA-SO_3_H (0.08 g, 0.64 mol%), H_2_O : EtOH (1 : 1, 2 mL), at room temperature.

b Isolated yield.

#### 3.2.3. Reaction mechanism

**Scheme 4:**
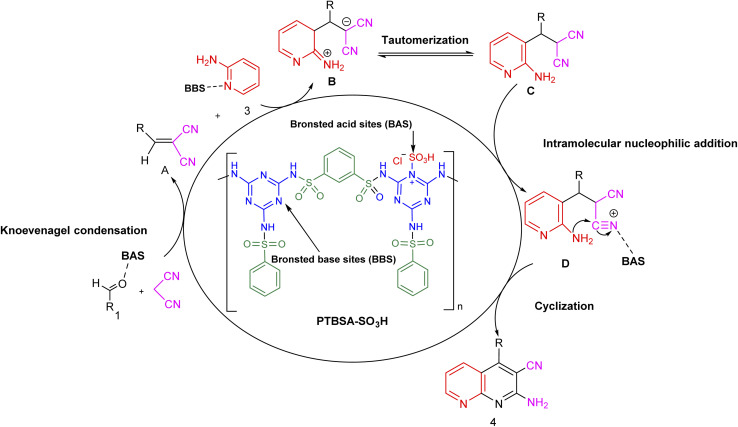
Suggested mechanism for the synthesis of 1,8-naphthyridines using PTBSA-SO_3_H.

Scheme 4 shows the mechanism offered for one-pot preparation of 1,8-naphthyridines through a three-component domino reaction catalyzed by PTBSA-SO_3_H. According to this figure, initially, PTBSA-SO_3_H catalyst enhances the activity of benzaldehyde and the Knoevenagel condensation of aldehyde 1 with malononitrile leads to the formation of intermediate A. Further, the next step involves nucleophilic attack of 2-aminopyridine (3) with intermediate A produces the intermediate B. In the final step, as it is evident in intermediate D, tautomerization, proton-transfer, oxidation and sequential intramolecular nucleophilic addition furnish the corresponding product.^47^

#### 3.2.4. Stability and reusability tests

**Fig. 10:**
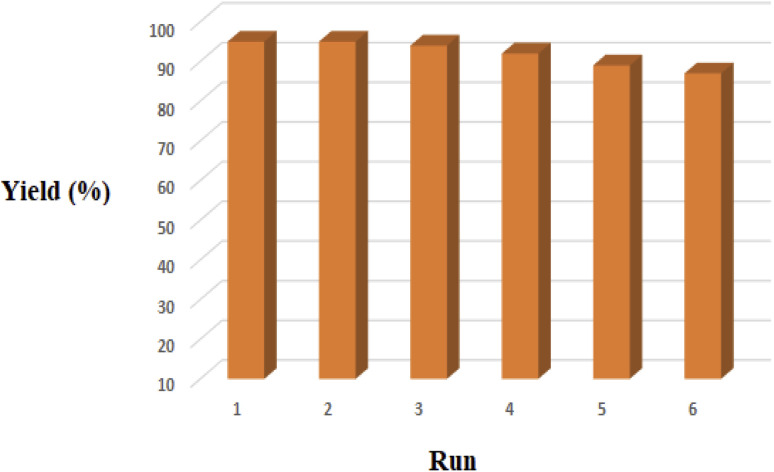
Recycling of the PTBSA-SO_3_H for the reaction of benzaldehyde, 2-aminopyridine and malononitrile.

**Fig. 11:**
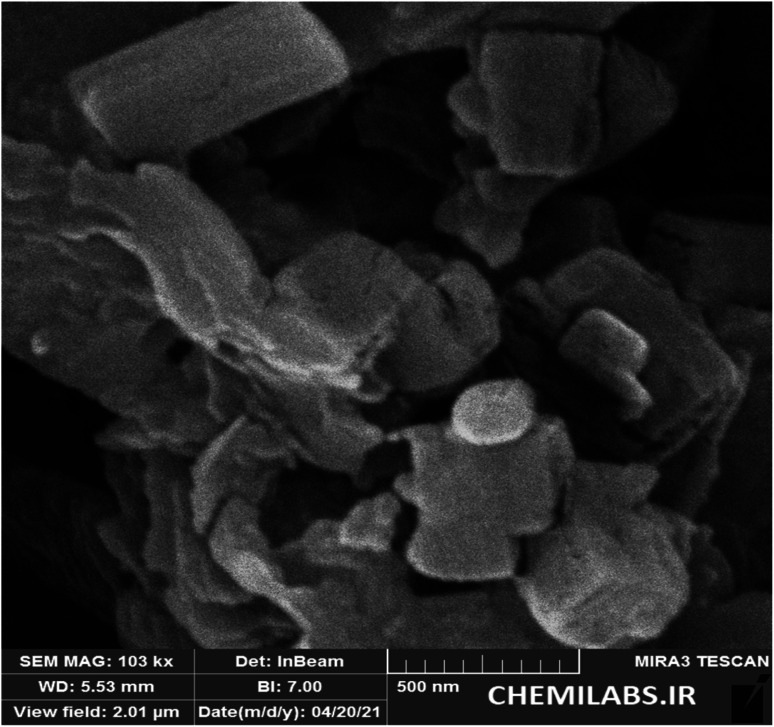
The FE-SEM image of PTBSA-SO_3_H after 6 runs.

One main aspect for the any catalyst, is the capability of the recovery and reusability. For this purpose, after completion of the reaction, the PTBSA-SO_3_H was recovered and its catalytic activity in the model reaction was studied that results shown this nanocatalyst can be recovered and reused for the six times with maintaining its catalytic activity, (95, 95, 94, 92, 89, 87) (Fig. 10). In addition, the FESEM image of recycled catalyst show very high stability of porous PTBSA-SO_3_H (Fig. 11).

#### 3.2.5. Comparison

Table 4 compares the catalytic performance of PTBSA-SO_3_H for those reported in the literature for the model reaction. As can be seen, the previous catalysts suffered from multiple disadvantages, including using dangerous reagents, non-renewable catalysts, temperature rise, and high amounts of catalysts. However, the prepared PTBSA-SO_3_H catalyst has a competing catalytic activity with those reported in the literature but without the mentioned shortcomings (entry 5).

**Table 4:** Comparison of the present methodology with other reported catalyst for the synthesis of 2-amino-4-phenyl-1,8-naphthyridine-3-carbonitrile (4a)

Entry	Catalyst	Solvent	Conditions	Time	Yield^a^ (%)	Ref
1	TBBDA	CH_3_CN	R.T.	3 h	87	48
2	PBBS	CH_3_CN	R.T.	3.5 h	80	49
3	Er/IDA/CPTMS@CoFe_2_O_4_	H_2_O	80 °C	6 min	92	50
4	Bi (NO_3_)_3_·5H_2_O	Solvent-free	MW, 160 °C	5 min	92	51
5	PTBSA-SO_3_H	H_2_O/EtOH (1 : 1)	R.T.	30 min	95	This work

a Isolated yield.

## 4. Conclusion

This work reports the cost-effective and sustainable synthesis of a novel polymer-based bifunctional catalyst supported cholorosulfonic acid [poly(triazine-benzene sulfonamide)*-*SO_3_H (PTBSA-SO_3_H)]. Significantly, a green and economical procedure was applied for accelerating the synthesis of 1,8-naphthyridine derivatives using various aryl aldehydes with a high variety of structural differences. The presented method is the most efficient catalytic system for the mentioned syntheses as compared to other synthetic methodologies. The catalyst showed high recyclability for more than six cycles without a significant loss in its catalytic activity. The recycled catalyst was characterized using SEM, and FT-IR, which revealed the high stability of the catalyst under optimal reaction conditions. Thus, this work can be regarded as highly significant in preparing inexpensive and sustainable catalytic systems using available materials.

## Conflicts of interest

No potential conflict of interest was reported by the author(s).

## Supplementary Material

RA-012-D2RA05070F-s001
