# Correction: Chlorosulfonic acid coated on porous organic polymer as a bifunctional catalyst for the one-pot three-component synthesis of 1,8-naphthyridines

**DOI:** 10.1039/d4ra90124j

**Published:** 2024-10-14

**Authors:** Ramin Ghiai, Sedigheh Alavinia, Ramin Ghorbani-Vaghei

**Affiliations:** a Department of Organic Chemistry, Faculty of Chemistry, Bu-Ali Sina University Hamedan 6517838683 Iran rgvaghei@yahoo.com ghorbani@basu.ac.ir +98 81 38380647

## Abstract

Correction for ‘Chlorosulfonic acid coated on porous organic polymer as a bifunctional catalyst for the one-pot three-component synthesis of 1,8-naphthyridines’ by Ramin Ghiai *et al.*, *RSC Adv.*, 2022, **12**, 27723–27735, https://doi.org/10.1039/D2RA05070F.

The authors regret that in Fig. 2, the FE-SEM of the starting material PTBSA in Fig. 2B is the same image the authors previously presented in ref. 5 of the original paper without being correctly attributed. The same starting material was used in both papers, so there are no changes to the discussions and conclusions presented, but the caption should have been as follows:

**Fig. 2** FE-SEM photographs of **PTBSA** (a) and (b) (b reproduced from ref. 5), **PTBSA-SO**_**3**_**H** (c) and (e) and particle size distribution of **PTBSA-SO**_**3**_**H** (f).

The Royal Society of Chemistry apologises for these errors and any consequent inconvenience to authors and readers.

**Reference**

5. A. Gharehkhani, R. Ghorbani-vaghei and S. Alavinia, *RSC Adv.*, 2021, **11**, 37514–37527.

