# Supplementary material for: Chlorosulfonic acid coated on porous organic polymer as a bifunctional catalyst for the one-pot three-component synthesis of 1,8-naphthyridines
Source: RSC Adv. 2022 Sep 28;12(43):27723–35. doi: 10.1039/d2ra05070f (PMC9516894; doi:10.1039/d2ra05070f)
Supplement: RA-012-D2RA05070F-s001 [file RA-012-D2RA05070F-s001.pdf]

Electronic Supplementary Material (ESI) for RSC Advances.
This journal is © The Royal Society of Chemistry 2022

Chlorosulfonic acid coated on porous organic polymer as a bifunctional catalyst for one-pot three-component synthesis of 1,8-naphthyridines

Supporting Information

Ramin Ghiai, Sedigheh Alavinia, Ramin Ghorbani-Vaghei*

Department of Organic Chemistry, Faculty of Chemistry, Bu-Ali Sina University, Hamedan, 6517838683, Iran

*Corresponding author; E-mail: rgvaghei@yahoo.com; Tel: +98(81)38380647

Spectral data for products.

2-Amino-4-phenyl-1,8-naphthyridine-3-carbonitrile

^{1}H NMR (300 MHz, DMSO-d_6) δ 8.52 (d, J = 7.9 Hz, 1H), 8.35 (s, 1H), 8.10–7.88 (m, 3H), 7.65 (d, J = 9.1 Hz, 1H), 7.58 (dd, J = 8.5, 6.5 Hz, 2H), 7.38–7.32 (m, 1H), 7.15 (ddd, J = 9.1, 6.5, 2.5 Hz, 1H), 6.80 (d, J = 6.8, 1.5 Hz, 1H). ^{13}C NMR (75 MHz, DMSO-d_6) δ 145.49, 144.21, 134.38, 133.91, 129.17, 128.16, 127.28, 126.01, 125.40, 116.98, 112.53, 109.74, 87.14.

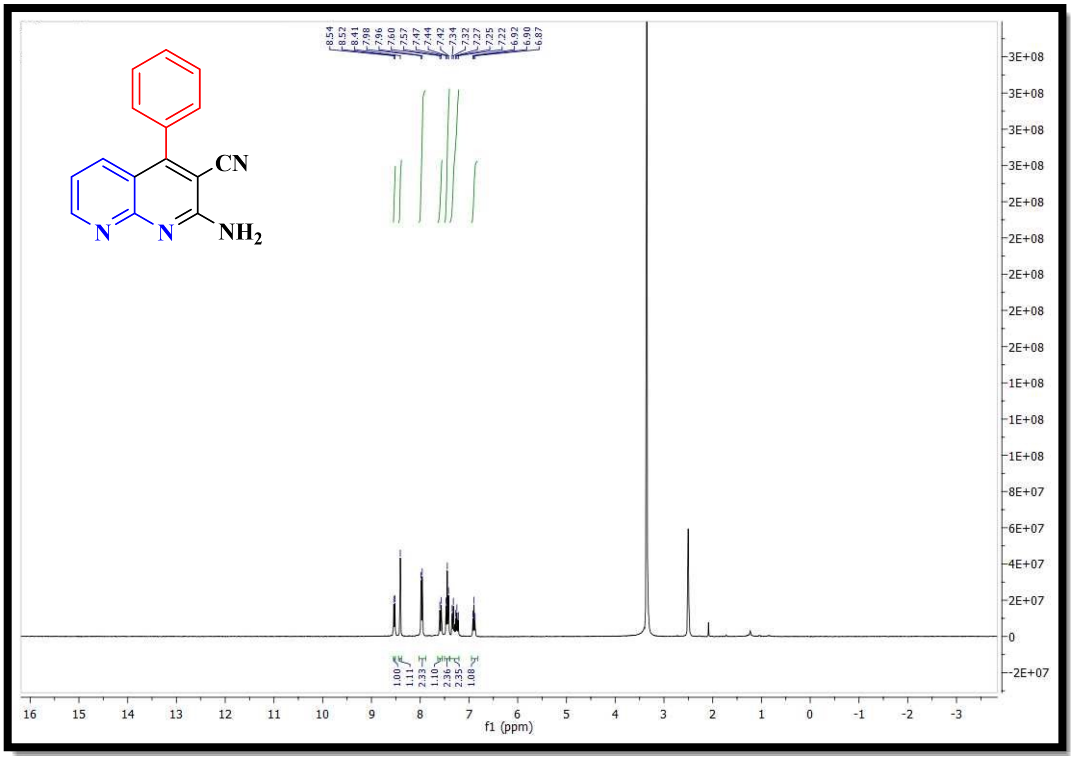

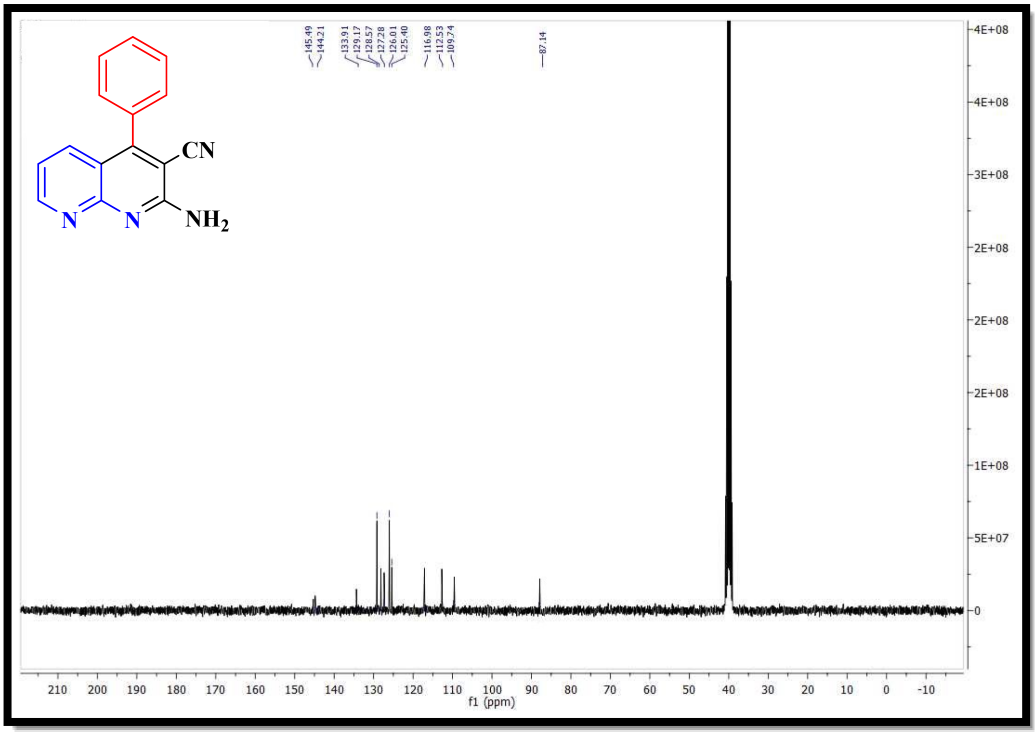

2-Amino-4-(4-nitrophenyl)-1,8-naphthyridine-3-carbonitrile

^{1}H NMR (500 MHz, DMSO-d$_6$) δ 8.88 (s, 2H), 8.82 (d, *J* = 8.8 Hz, 2H), 8.51 (d, *J* = 8.6 Hz, 2H), 8.19 (d, *J* = 8.2 Hz, 2H), 7.88 (d, *J* = 8.2 Hz, 1H), 7.80 (d, *J* = 8.5 Hz, 1H), 7.37 (d, *J* = 7.9 Hz, 1H). ^{13}C NMR (126 MHz, DMSO-d$_6$) δ 158.58, 156.60, 148.02, 142.43, 136.29, 135.40, 132.33, 128.70, 127.37, 124.77, 116.88, 114.15, 113.96, 112.94, 87.33.

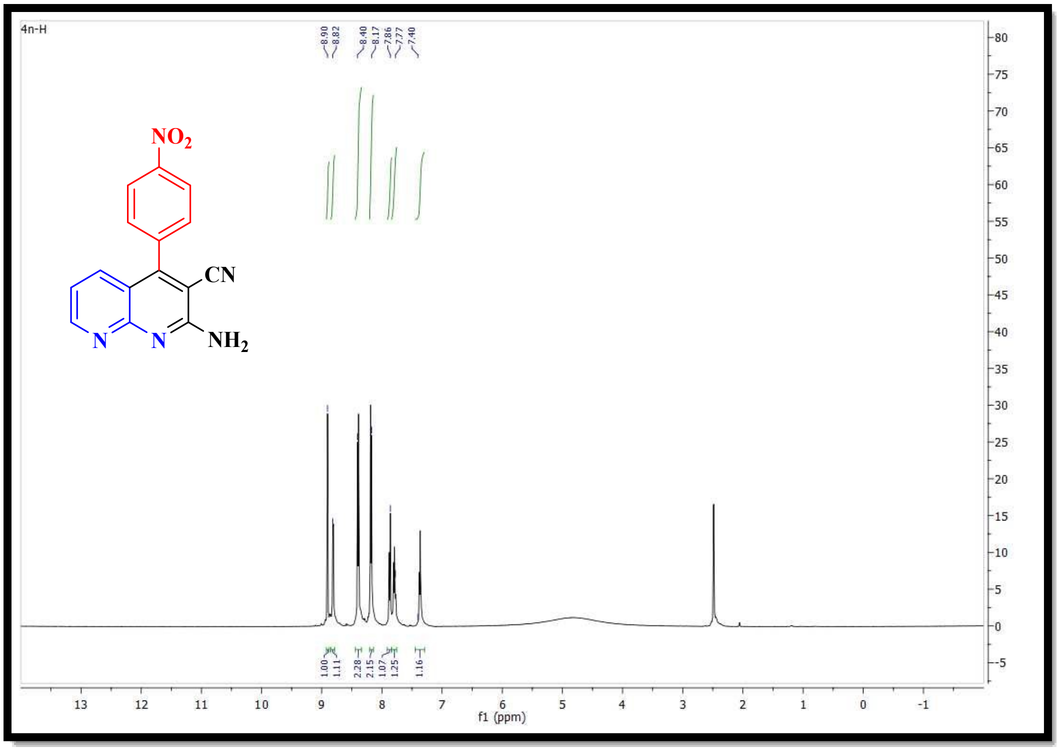

4n-H
NO_2
CN
N
N
NH_2
8.90
8.82
8.40
8.17
7.86
7.77
7.40
1.00
1.11
2.28
2.15
1.07
1.25
1.16
f1 (ppm)

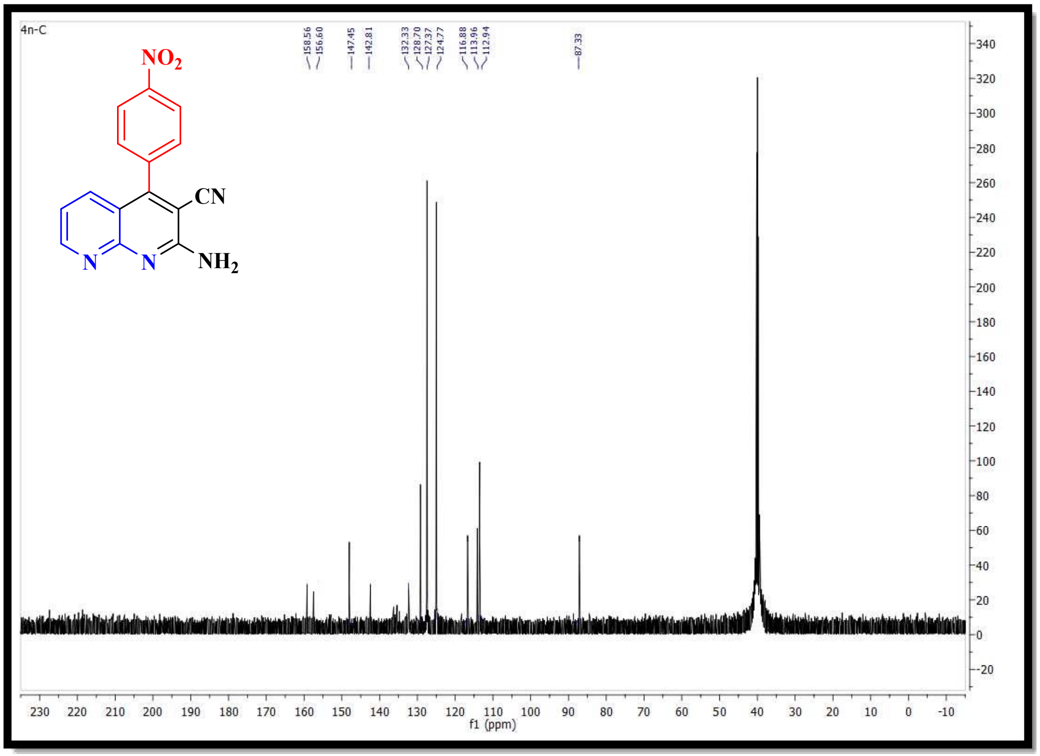

4n-C
NO_2
CN
N
N
NH_2
158.56
156.60
147.45
142.81
132.33
128.70
127.37
124.77
116.88
113.96
112.94
87.33
f1 (ppm)

2-Amino-4-(2-chlorophenyl)-1,8-naphthyridine-3-carbonitrile

^{1}H NMR (500 MHz, DMSO-d_6) δ 8.52 (d, J = 7.7 Hz, 1H), 8.49 (s, 1H), 8.00 (t, J = 2.9 Hz, 1H), 7.95–7.89 (m, 1H), 7.58 (d, J = 8.5 Hz, 1H), 7.46 (t, J = 8.8 Hz, 1H), 7.36 (dd, J = 8.9, 2.5 Hz, 1H), 7.31–7.22 (m, 1H), 6.90 (t, J = 6.7 Hz, 1H). ^{13}C NMR (126 MHz, DMSO-d_6) δ 159.59, 144.77, 143.13, 136.26, 128.08, 127.05, 126.42, 125.77, 124.13, 117.51, 112.62, 110.66, 87.33.

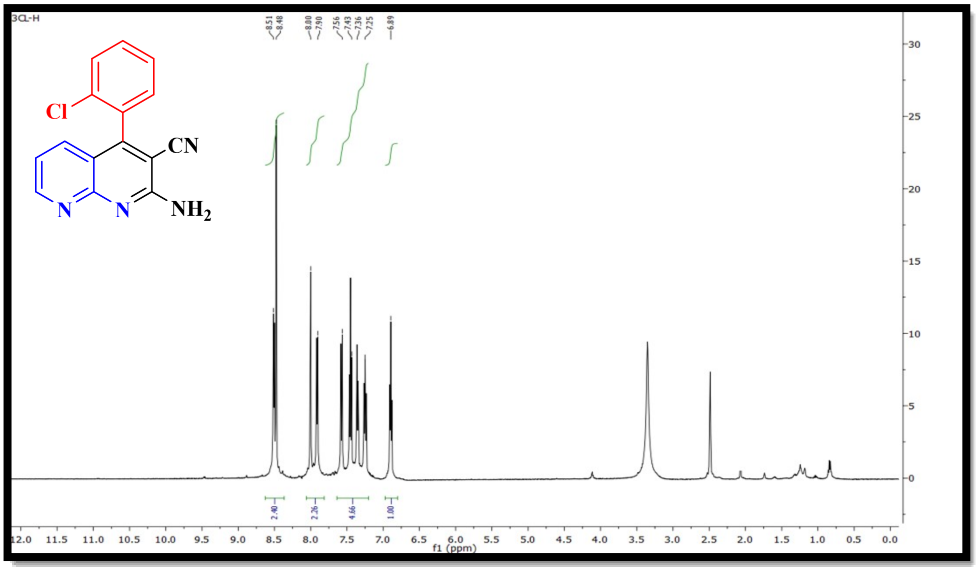

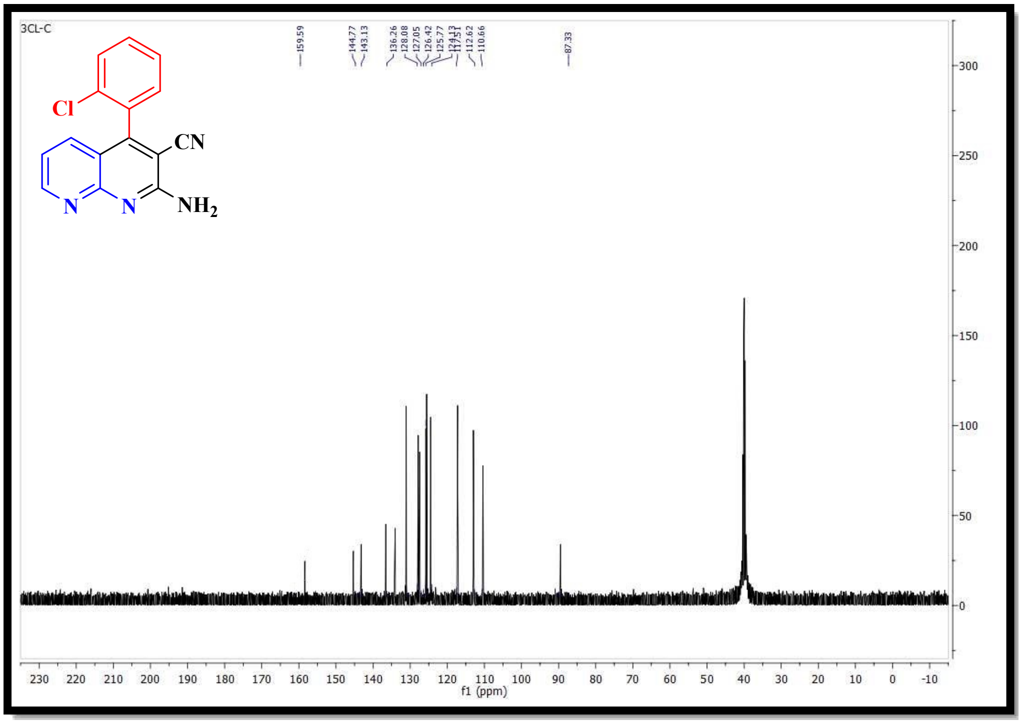

2-Amino-4-(4-bromoophenyl)-1,8-naphthyridine-3-carbonitrile

^{1}H NMR (500 MHz, DMSO-d$_6$) δ 8.88 (d, *J* = 7.8 Hz, 1H), 8.85 (s, 2H), 7.93 (d, *J* = 6.5 Hz, 1H), 7.84 (d, *J* = 8.5 Hz, 2H), 7.79 (d, *J* = 8.5 Hz, 2H), 7.50 (d, *J* = 6.5, 3.5 Hz, 1H). ^{13}C NMR (126 MHz, DMSO-d$_6$) δ 157.31, 154.63, 140.72, 134.89, 134.09, 133.93, 129.43, 128.46, 126.34, 124.18, 117.72, 112.69, 112.18, 109.99, 85.68.

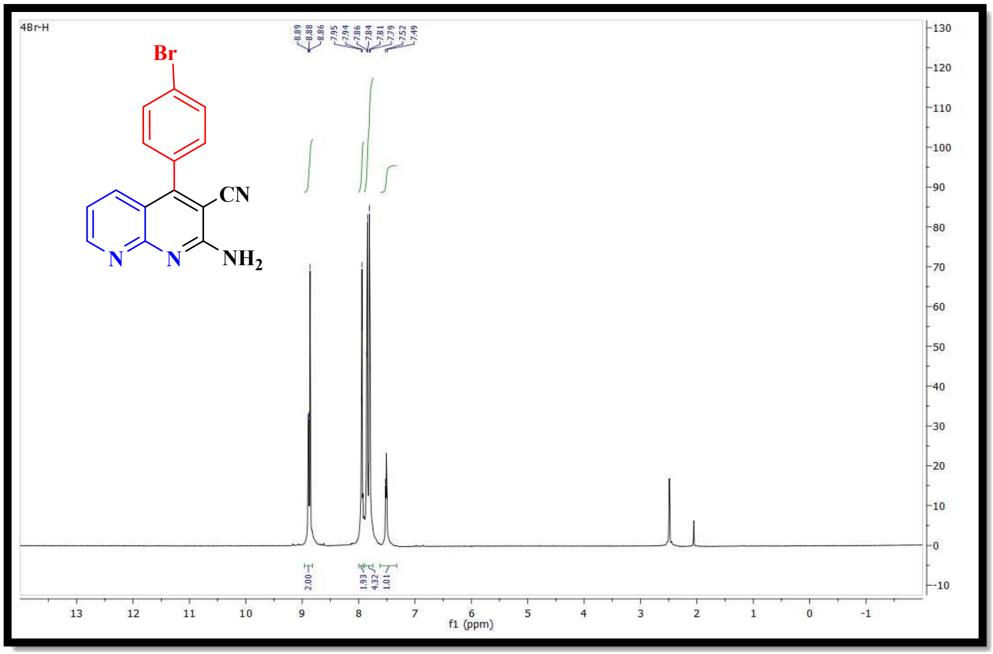
4Br-H
Br
CN
N
N
NH2
8.89
8.88
8.86
7.95
7.94
7.86
7.84
7.81
7.79
7.52
7.49
2.00
1.93
4.32
1.01
f1 (ppm)

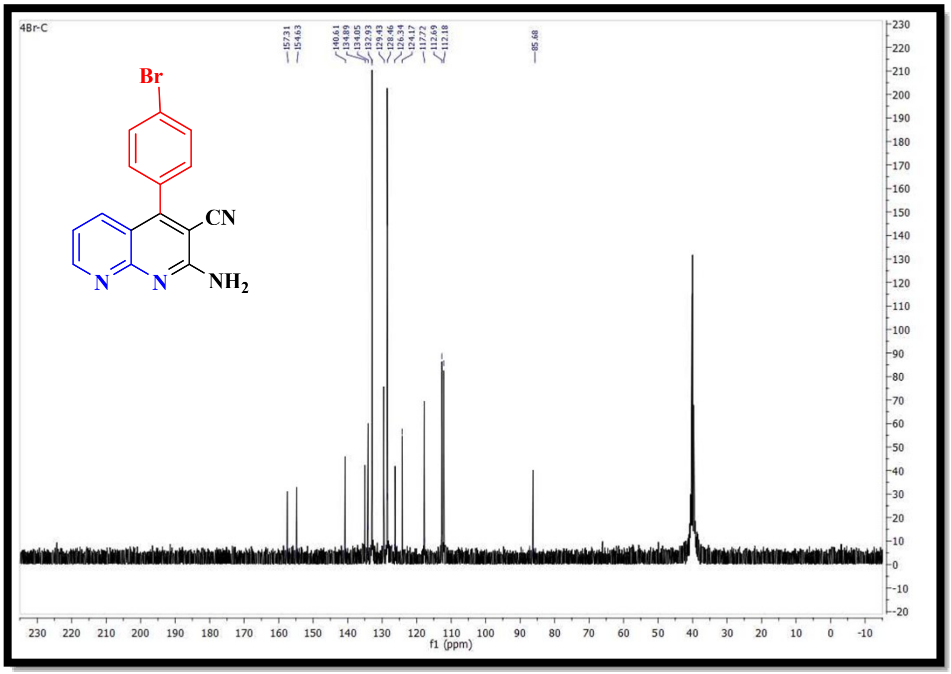
4Br-C
Br
CN
N
N
NH2
157.31
154.63
140.61
134.89
134.05
132.93
129.43
128.46
126.34
124.17
117.72
112.69
112.18
85.68
f1 (ppm)

2-Amino-4-(4-chlorophenyl)-1,8-naphthyridine-3-carbonitrile

^{1}H NMR (500 MHz, DMSO-d$_6$) δ 8.52 (d, *J* = 6.8 Hz, 1H), 8.34 (s, 2H), 7.98 –7.95 (m, 1H), 7.64 (d, *J* = 9.3 Hz, 1H), 7.55–7.48 (m, 1H), 7.34-7.22 (m, 1H), 6.91 (t, *J* = 7.8 Hz, 1H). ^{13}C NMR (126 MHz, DMSO-d$_6$) δ 157.94, 154.63, 144.78, 142.81, 132.96, 129.33, 127. 71, 125.79, 124.45, 116.88, 112.64, 110.03, 87.64.

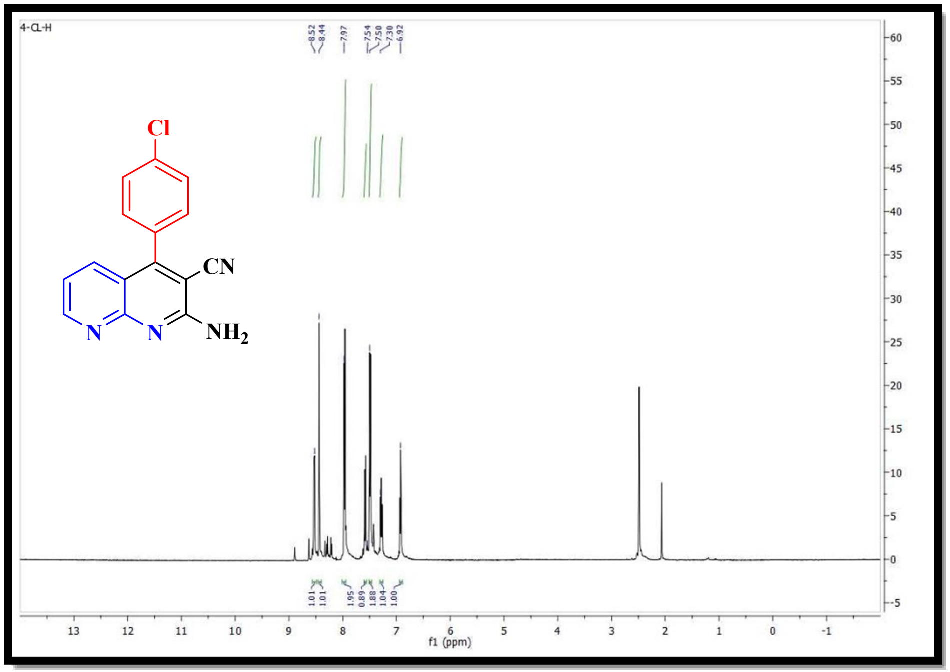

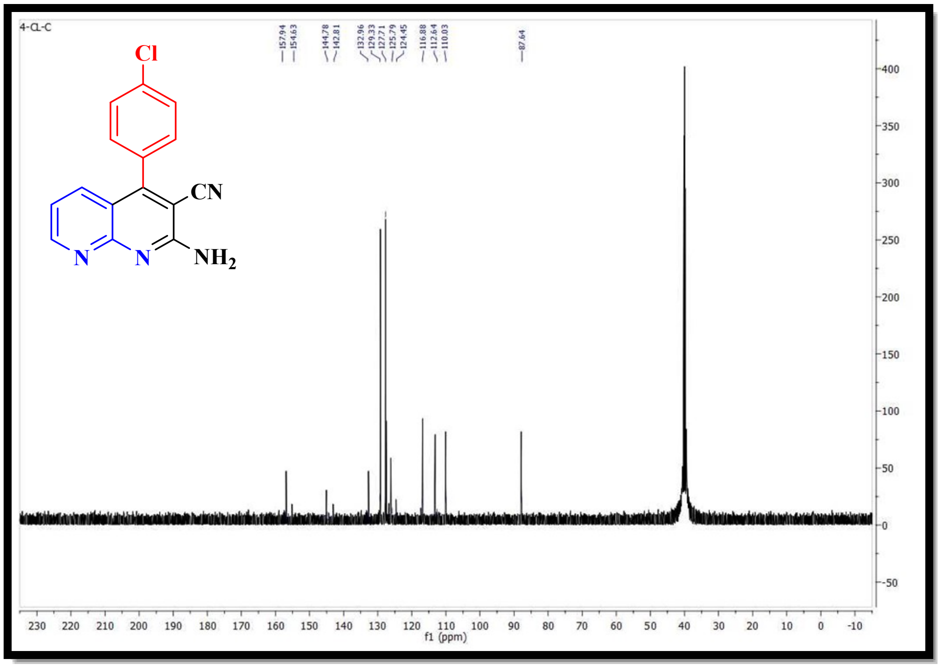

2-Amino-4-(3-nitrophenyl)-1,8-naphthyridine-3-carbonitrile

^{1}H NMR (500 MHz, DMSO-d$_6$) δ 8.79 – 8.74 (m, 1H), 8.62 (s, 1H), 8.56 (d, *J* = 6.8 Hz, 1H), 8.39 (d, *J* = 7.5 Hz, 1H), 8.15 (ddd, *J* = 8.5, 2.5, 1.5 Hz, 1H), 7.74 (t, *J* = 8.0 Hz, 1H), 7.65–7.59 (m, 1H), 7.30 (ddd, *J* = 9.2, 7.7, 2.5 Hz, 1H), 6.94 (d, *J* = 6.8 Hz, 1H). ^{13}C NMR (126 MHz, DMSO-d$_6$) δ 158.26, 153.37, 148.85, 145.47, 142.43, 136.19, 132.64, 130.99, 127.05, 126.41, 122.78, 119.88, 117.50, 113.26, 110.99, 84.02.

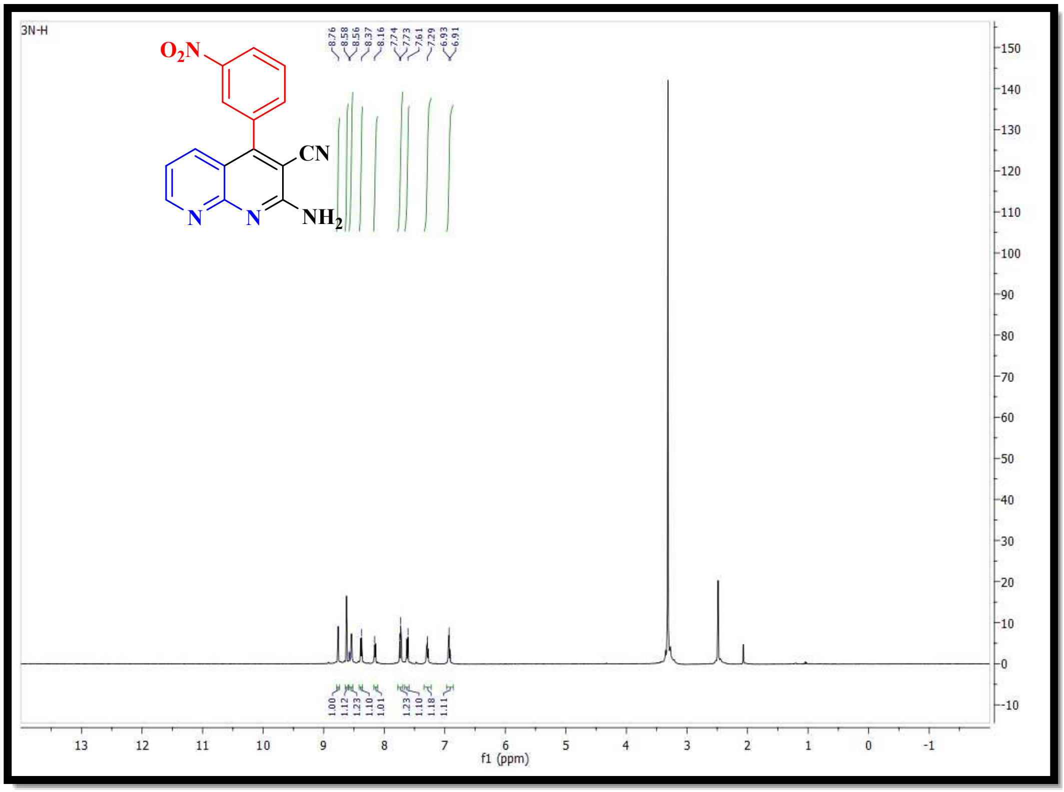
3N-H
O_2N
CN
N
N
NH_2
8.76
8.58
8.56
8.37
8.16
7.74
7.73
7.61
7.29
6.93
6.91
1.00
1.12
1.23
1.10
1.01
1.23
1.10
1.18
1.11
f1 (ppm)

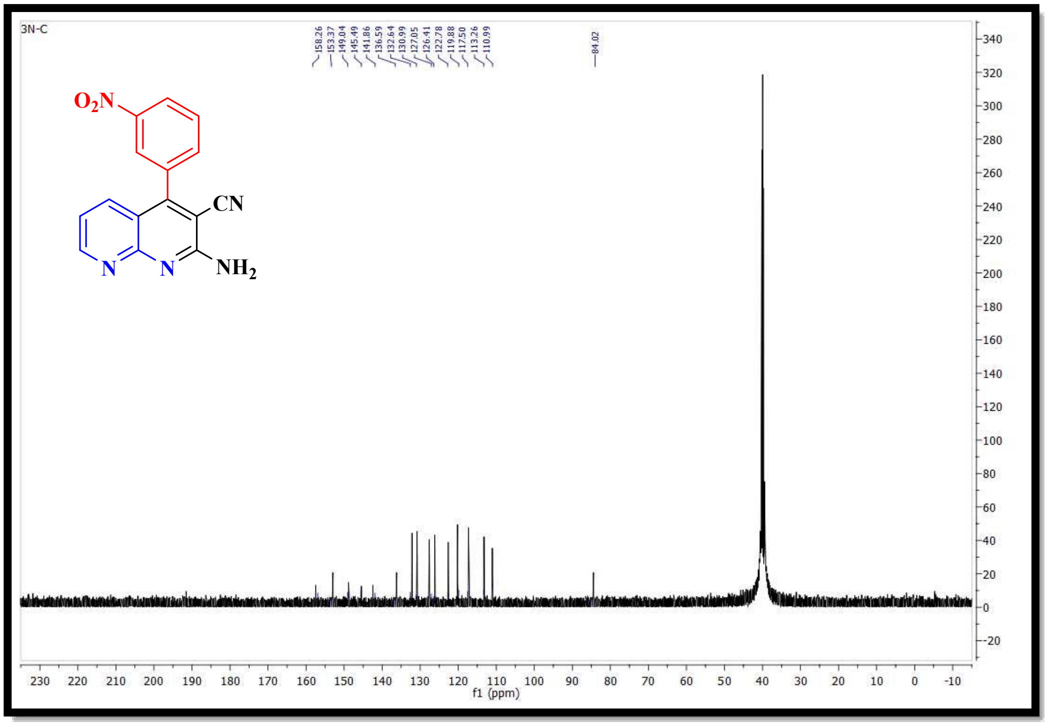
3N-C
O_2N
CN
N
N
NH_2
158.26
153.37
149.04
145.49
141.86
136.59
132.64
130.99
127.05
126.41
122.78
119.88
117.50
113.26
110.99
84.02
f1 (ppm)

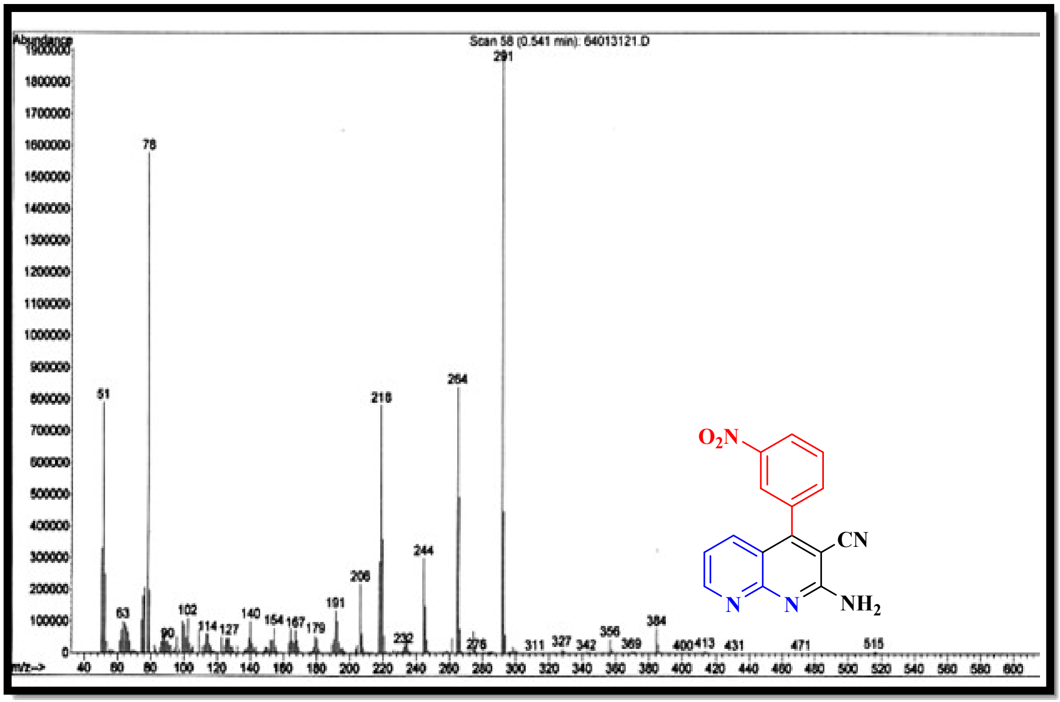

2-Amino-4-(4-methoxyphenyl)-1,8-naphthyridine-3-carbonitrile

^{1}H NMR (300 MHz, DMSO-d$_6$) δ 8.78 (d, *J* = 7.7 Hz, 1H), 8.75 – 8.55 (m, 4H), 7.99–7.70 (m, 6H), 7.60 (d, *J* = 7.5, 4.6 Hz, 1H), 7.29 (d, *J* = 8.4 Hz, 3H), 3.85 (s, 3H). ^{13}C NMR (75 MHz, DMSO-d$_6$) δ 159.32, 148.50, 138.20, 135.45, 128.37, 128.26, 120.30, 117.73, 115.16, 112.51, 110.95, 80.12, 56.98.

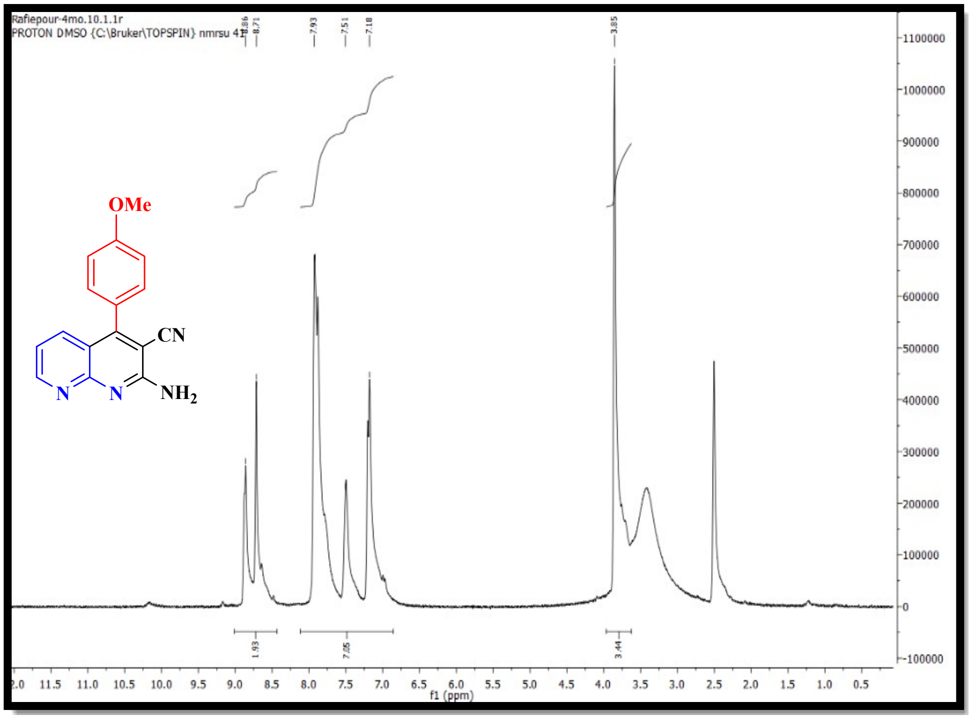

2-Amino-4-(4-hydroxyphenyl)-1,8-naphthyridine-3-carbonitrile

^{1}H NMR (500 MHz, DMSO-d$_6$) δ 10.10 (s, 1H), 8.92 – 8.80 (m, 1H), 8.62 (s, 1H), 7.97–7.85 (t, 2H), 7.75–7.65 (m, 1H), 7.59 (d, *J* = 7.5, 2.5 Hz, 1H), 6.88 – 6.93 (m, 1H). ^{13}C NMR (126 MHz, DMSO-d$_6$) δ 160.23, 139.90, 136.26, 133.28, 129.29, 128.70, 127.77, 117.73, 117.20, 111.99, 110.04, 86.39.

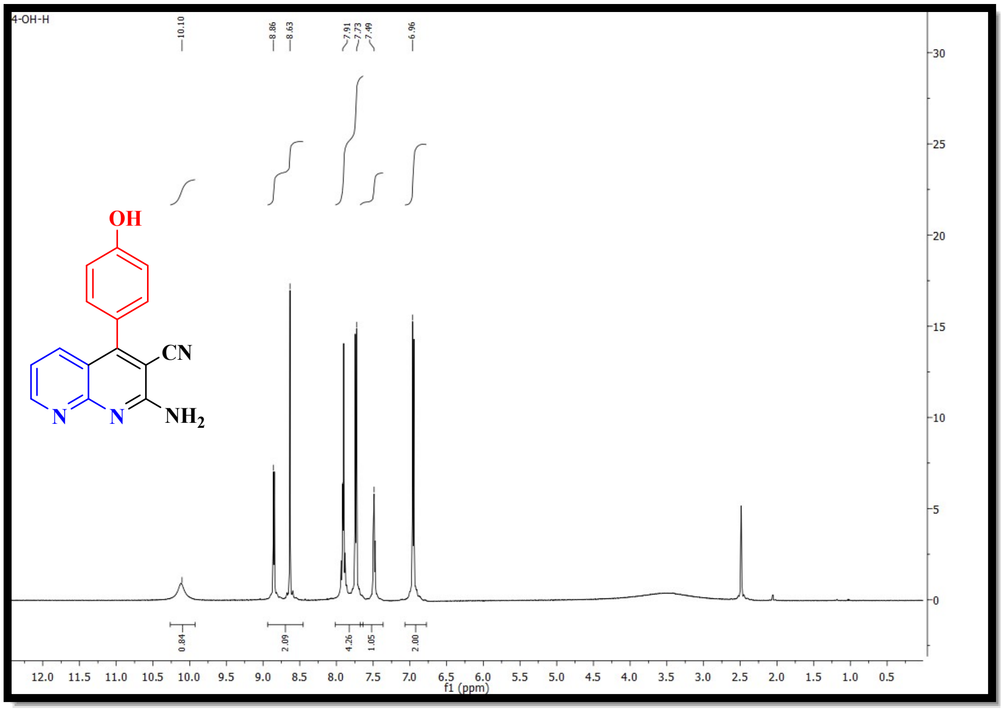
4-OH-H
10.10
8.86
8.63
7.91
7.73
7.49
6.96
OH
CN
N
N
NH2
0.84
2.09
4.26
1.05
2.00
f1 (ppm)

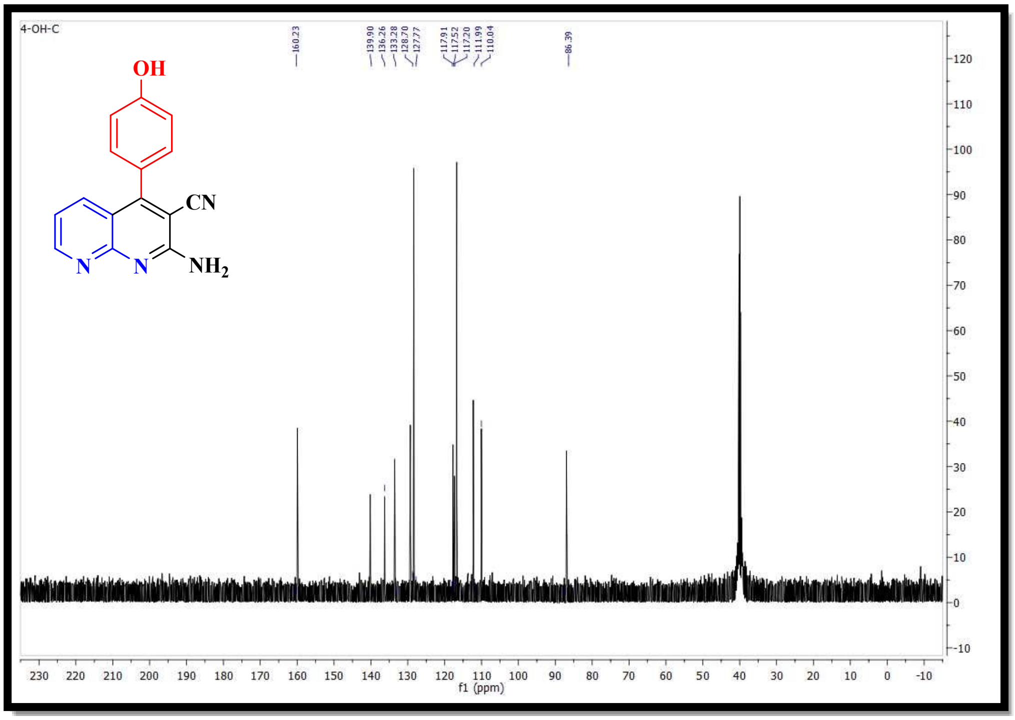
4-OH-C
160.23
139.90
136.26
133.28
128.70
127.77
117.91
117.52
117.20
111.99
110.04
86.39
OH
CN
N
N
NH2
f1 (ppm)

2-Amino-4-(*p*-tolyl)-1,8-naphthyridine-3-carbonitrile

^{1}H NMR (500 MHz, DMSO-d_6) δ 8.88 – 8.80 (m, 1H), 7.96 (ddd, *J* = 9.3, 6.8, 2.5 Hz, 2H), 7.77 (d, *J* = 9.5 Hz, 2H), 7.76 – 7.65 (m, 2H), 7.57 (dd, *J* = 6.5, 2.7 Hz, 1H), 2.07 (s, 3H). ^{13}C NMR (126 MHz, DMSO-d_6) δ 151.71, 139.45, 135.20, 133.42, 130.84, 130.85, 129.90, 127.45, 126.52, 120.30, 117.32, 112.46, 83.39, 9.19.

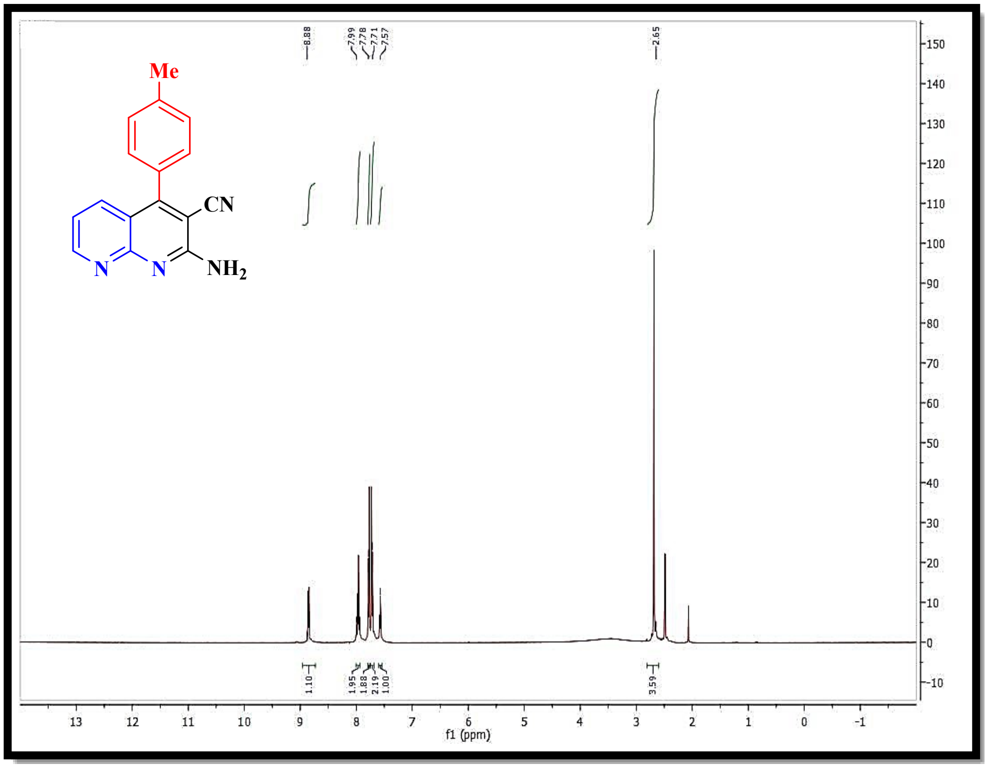

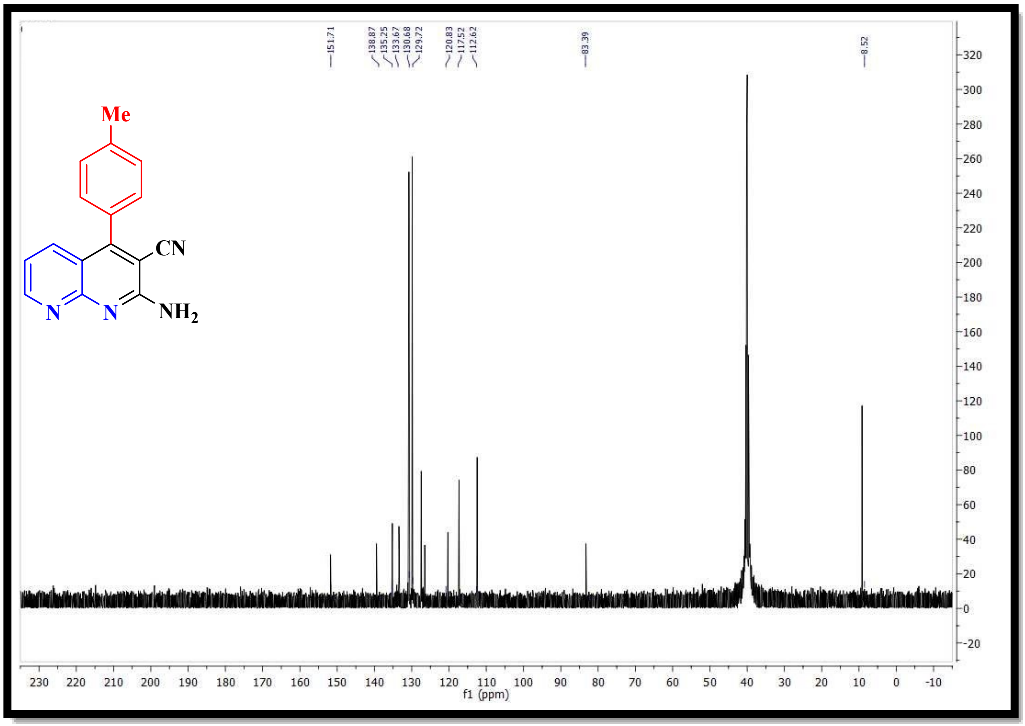

2-Amino-4-(biphenyl)-1,8-naphthyridine-3-carbonitrile

^{1}H NMR (500 MHz, DMSO-d_6) δ 8.64 (d, *J* = 7.9 Hz, 1H), 8.50 (d, *J* = 4.9 Hz, 2H), 8.12 (dd, *J* = 9.6, 2.6 Hz, 1H), 7.97 (t, *J* = 9.2 Hz, 2H), 7.83 (d, *J* = 7.5 Hz, 1H), 7.58 (d, *J* = 9.3 Hz, 1H), 7.51 (t, *J* = 15.0, 7.3, 3.9 Hz, 3H), 7.24 (t, *J* = 6.9 Hz, 1H), 6.80 (t, *J* = 7.8 Hz, 1H). ^{13}C NMR (126 MHz, DMSO-d_6) δ 157.31, 154.61, 147.45, 133.67, 132.64, 131.71, 128.39, 126.42, 125.39, 124.45, 117.19, 116.89, 112.95, 110.34, 80.48.

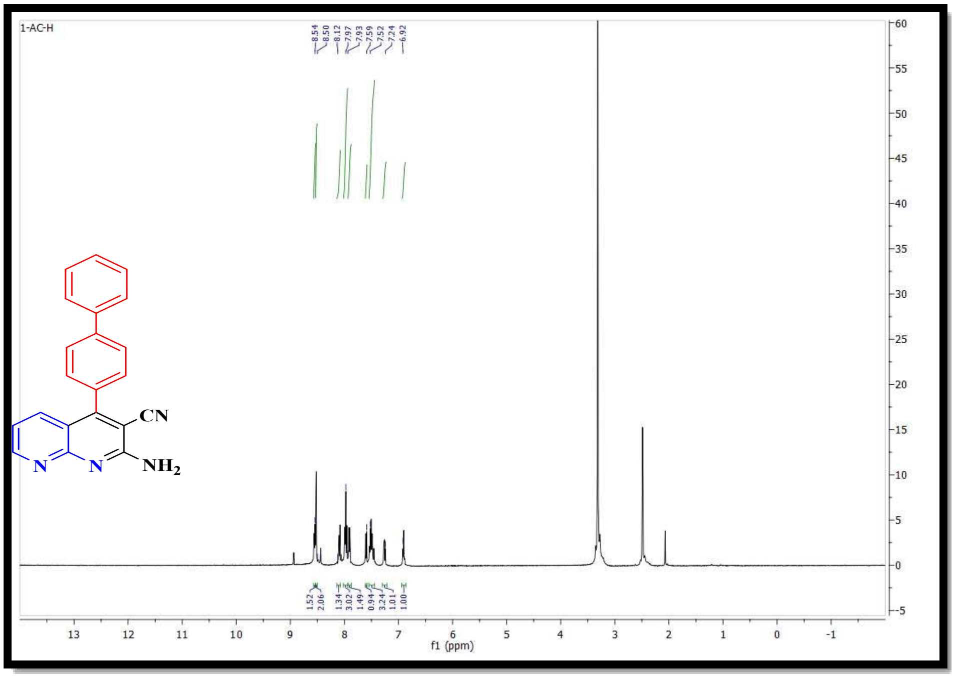
1-AC-H
8.54
8.50
8.12
7.97
7.93
7.59
7.52
7.24
6.92
CN
N
N
NH2
1.52
2.06
1.34
3.02
1.49
0.94
3.24
1.01
1.00
f1 (ppm)

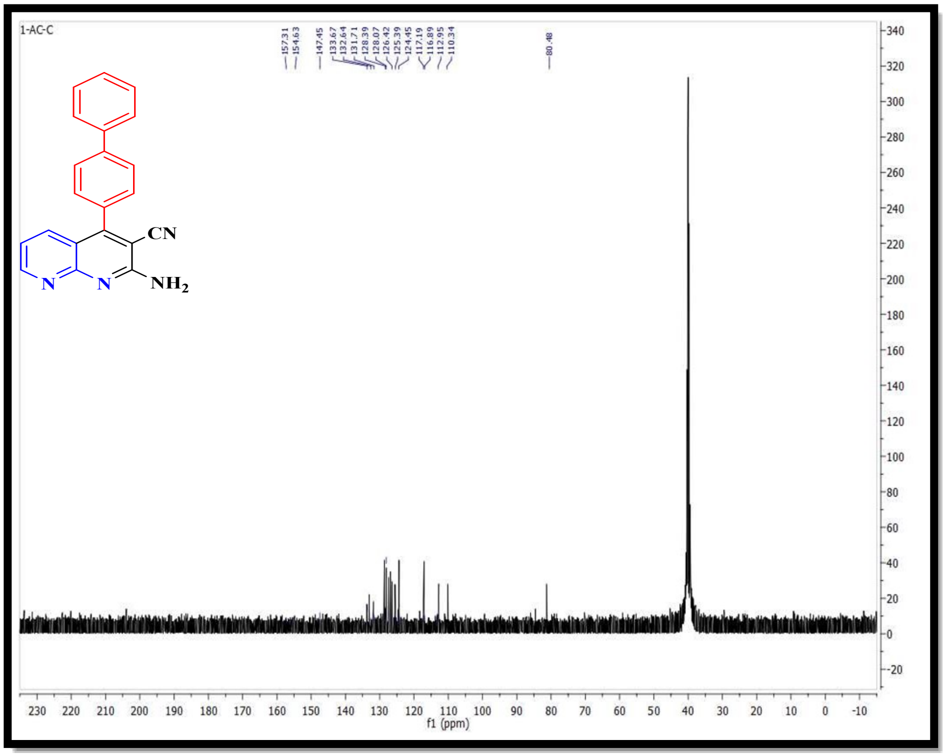
1-AC-C
157.31
154.63
147.45
133.67
132.64
131.71
128.39
128.07
126.42
125.39
124.45
117.19
116.89
112.95
110.34
80.48
CN
N
N
NH2
f1 (ppm)

2-Amino-4-(2-hydroxy-3-methoxyphenyl)-1,8-naphthyridine-3-carboxylate: ^{1}H NMR (400 MHz, DMSO-d_6): δ 3.34 (s, 1H, OH), 3.67 (s, 3H, OCH_3), 3.71 (s, 2H, NH_2), 3.85 (s, 3H, OCH_3), 6.56-6.59 (t, J = 4.8 Hz, 1H, ArH), 6.94-6.96 (d, J = 7.2 Hz, 1H, ArH), 7.05-7.18 (m, 3H, ArH), 7.92-7.94 (t, J = 3.2 Hz, 1H, ArH); ^{13}C NMR (100 MHz, DMSO-d_6): δ 55.9, 56.1, 108.0, 111.8, 116.2, 117.6, 119.2, 120.1, 122.5, 123.0, 124.4, 137.0, 139.1, 147.6, 148.3, 149.0, 159.7, 164.5.

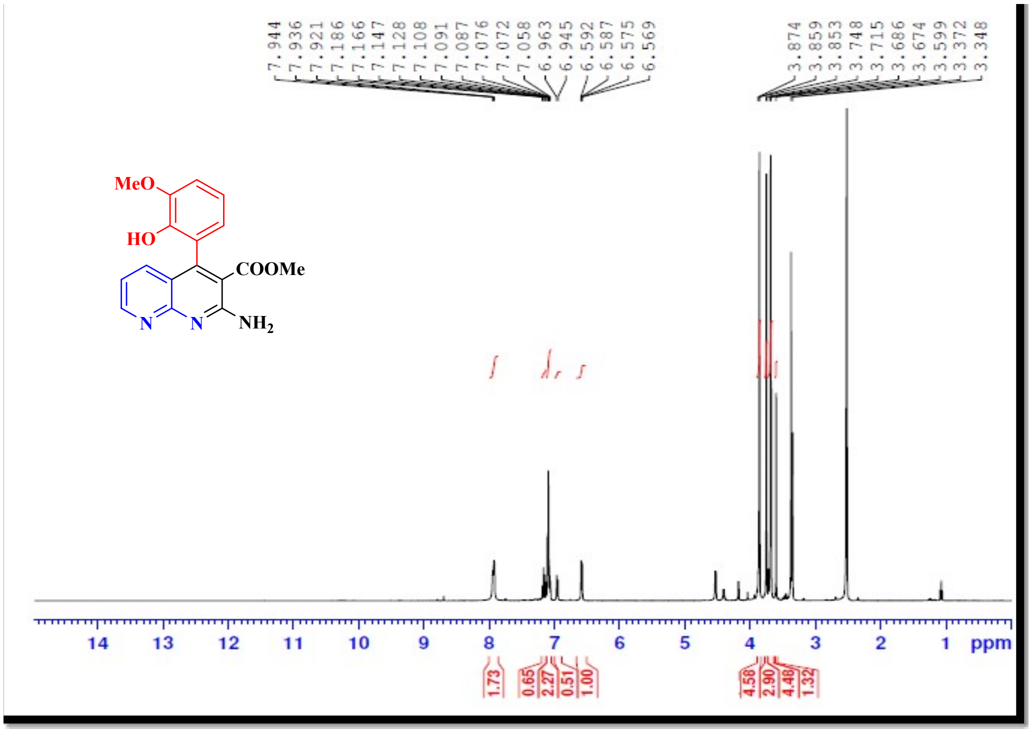

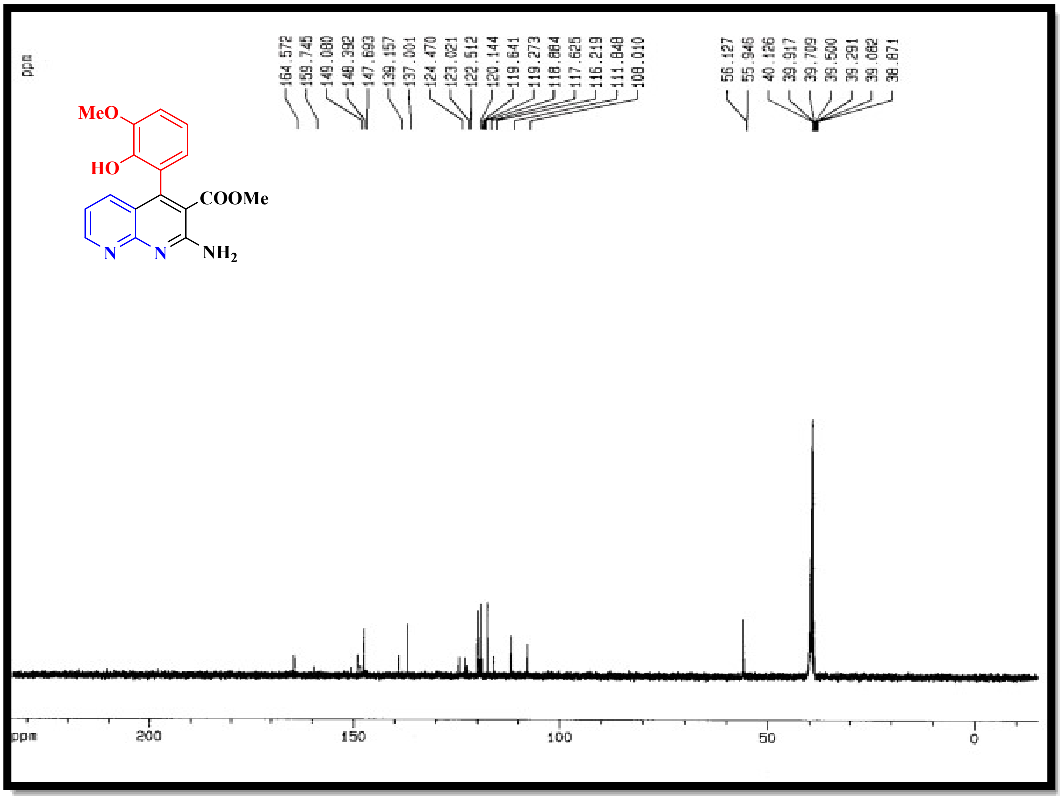

2-Amino-6-bromo-4-(5-bromo-2-hydroxyphenyl)-1,8-naphthyridine-3-carbonitrile:^{1}H NMR (90 MHz, $CDCl_3$): δ 6.86-7.49 (m, 5H, ArH, NH_2), 8.52 (s, 1H, ArH), 9.32 (s, 1H, ArH), 13.21 (s, 1H, OH); ^{13}C NMR (100 MHz, $CDCl_3$): δ_c: 109.7, 118.3, 119.2, 120.8, 134.4, 135.6, 140.0, 149.1, 154.7, 159.8, 162.8.

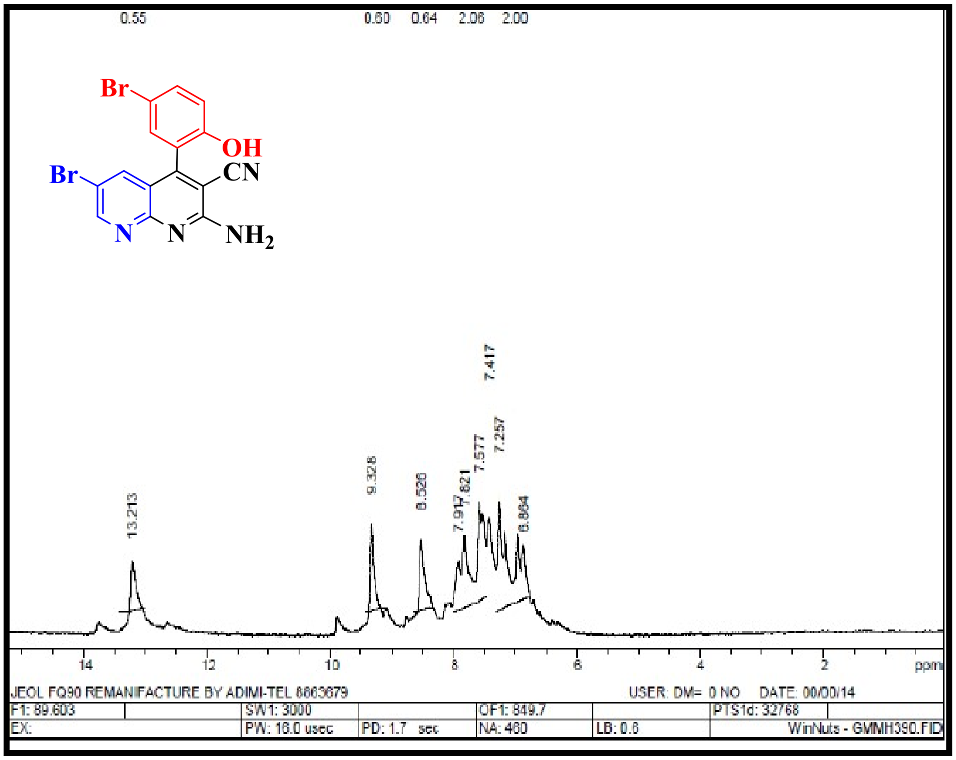
0.55
0.60 0.64 2.06 2.00
Br
OH
Br
CN
N
N
NH2
13.213
9.328
8.526
7.917
7.821
7.577
7.417
7.257
6.864
14
12
10
8
6
4
2
ppm
JEOL FQ90 REMANIFACTURE BY ADIMI-TEL 8863679
USER: DM= 0 NO DATE: 00/00/14
F1: 89.603
SW1: 3000
OF1: 849.7
PTS1d: 32768
EX:
PW: 18.0 usec
PD: 1.7 sec
NA: 480
LB: 0.8
WinNuts - GMMH390.FID

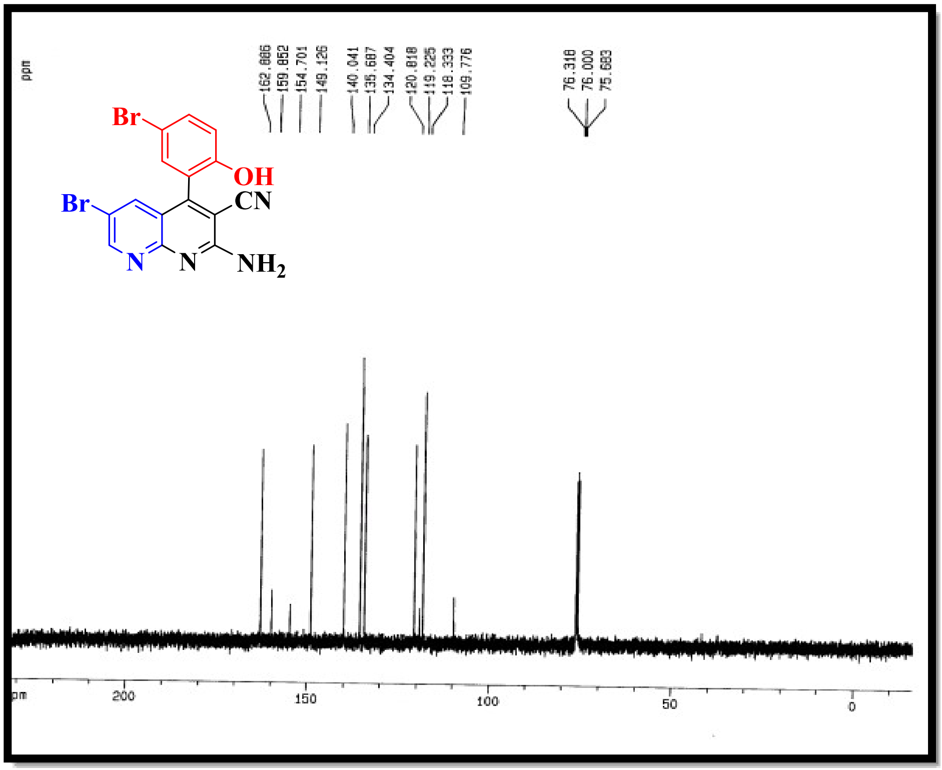
ppm
162.866
159.852
154.701
149.126
140.041
135.687
134.404
120.818
119.225
118.333
109.776
76.318
76.000
75.683
Br
OH
Br
CN
N
N
NH2
200
150
100
50
0

2-Amino-4-(2-hydroxy-3-methoxyphenyl)-1,8-naphthyridine-3-carbonitrile: ^{1}H NMR (400 MHz, $CDCl_3$): δ 3.90 (s, 3H, OCH_3), 7.02-7.04 (d, J = 8.0 Hz, 1H, ArH), 7.05-7.09 (t, J = 6.8 Hz, 1H, ArH), 7.15-7.19 (t, J = 5.6 Hz, 1H, ArH), 7.45-7.47 (d, J = 7.6 Hz, 1H, ArH), 7.50 (br, 2H, NH_2), 7.58-7.61 (d, J = 8.8 Hz, 1H, ArH), 7.81-7.82 (t, J = 4.0 Hz, 1H, ArH), 9.37 (d, J = 7.2 Hz, 1H, ArH); ^{13}C NMR (100 MHz, $CDCl_3$): δ 54.5, 110.5, 115.1, 115.6, 119.8, 125.6, 128.0, 128.8, 130.7, 137.9, 150.4, 152.4, 159.1, 161.2, 183.8.

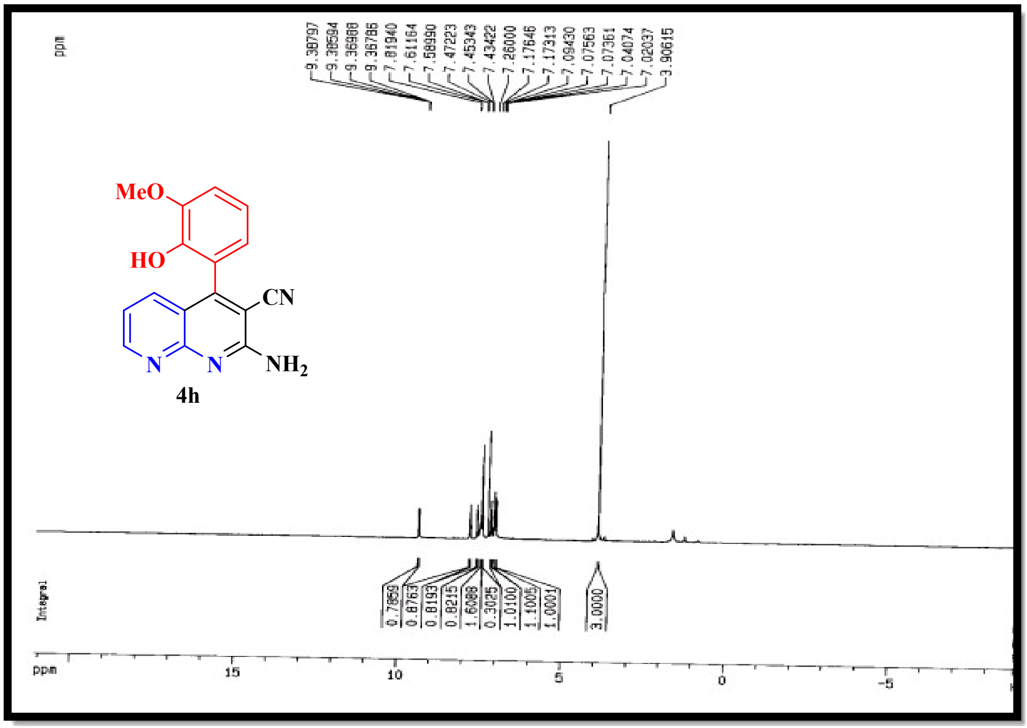

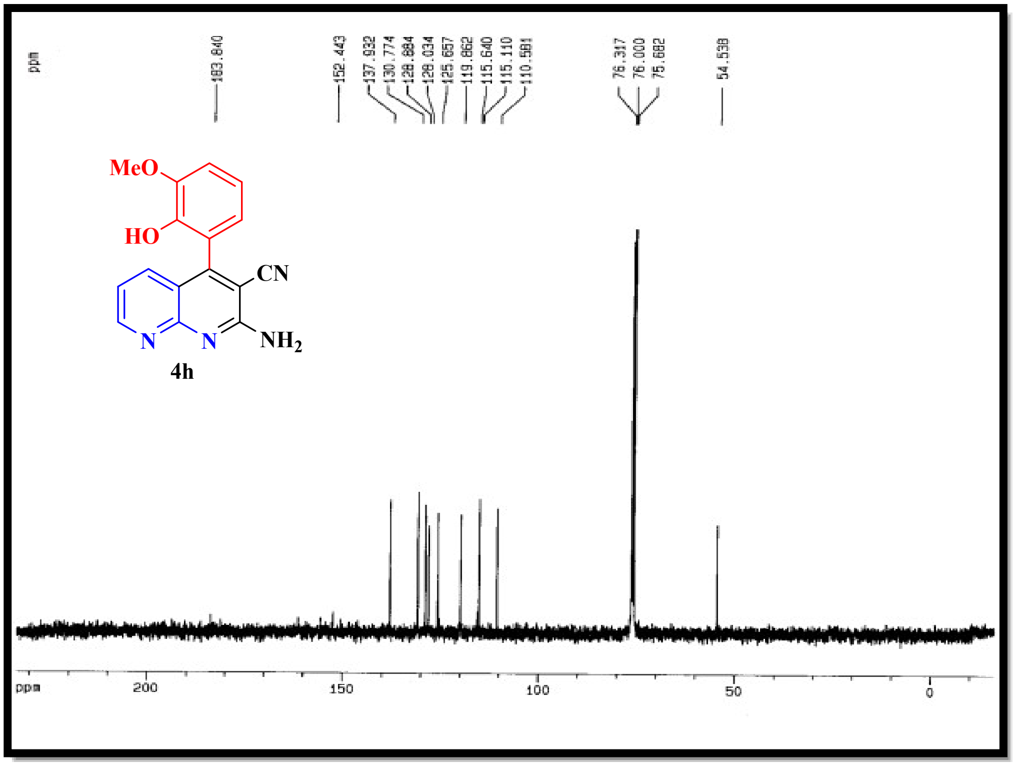

2-Amino-4-(pyridin-2-yl)-1,8-naphthyridine-3-carbonitrile:^{1}H NMR (400 MHz, DMSO-d_6): δ 7.65-7.68 (t, *J* = 6.8 Hz, 1H, ArH), 7.94-7.98 (t, 1H, *J* = 6.8 Hz, ArH), 8.03-8.07 (d, *J* = 10.0 Hz, 1H, ArH), 8.11-8.12 (d, 2H, NH_2), 8.38-8.40 (d, *J* = 8.4 Hz, 1H, ArH), 8.85-8.86 (d, *J* = 4.4 Hz, 1H, ArH), 9.14-9.16 (d, *J* = 7.2 Hz, 1H, ArH); ^{13}C NMR (75 MHz, DMSO-d_6): δ 108.0, 110.9, 113.3, 117.2, 118.4, 122.8, 124.4, 128.5, 129.7, 130.0, 137.3, 138.4, 142.7, 150.4, 152.8, 157.5.

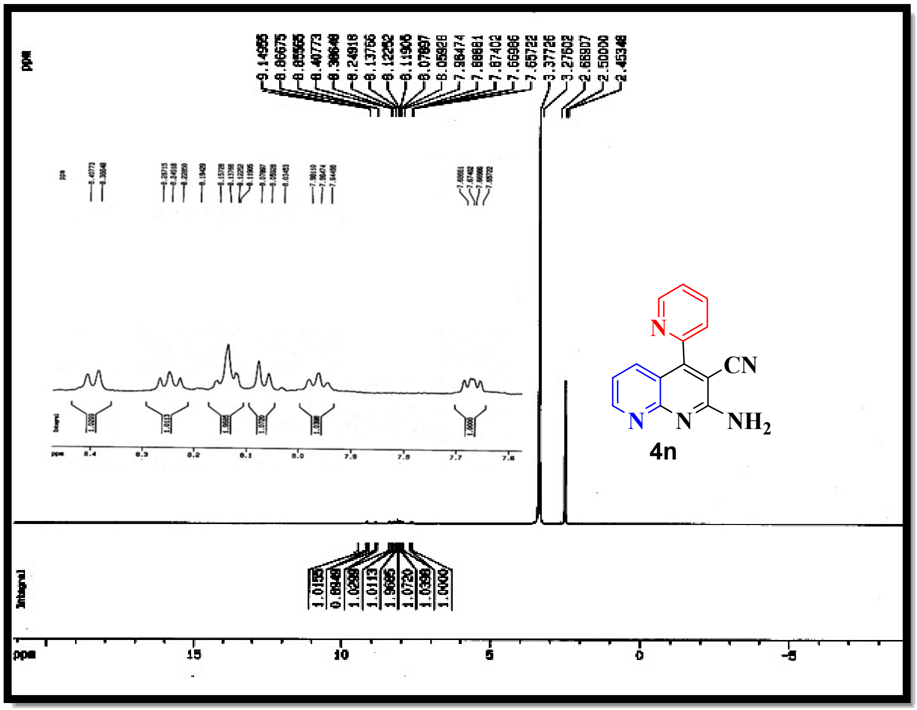
CN
NH2
4n
Integral
ppm

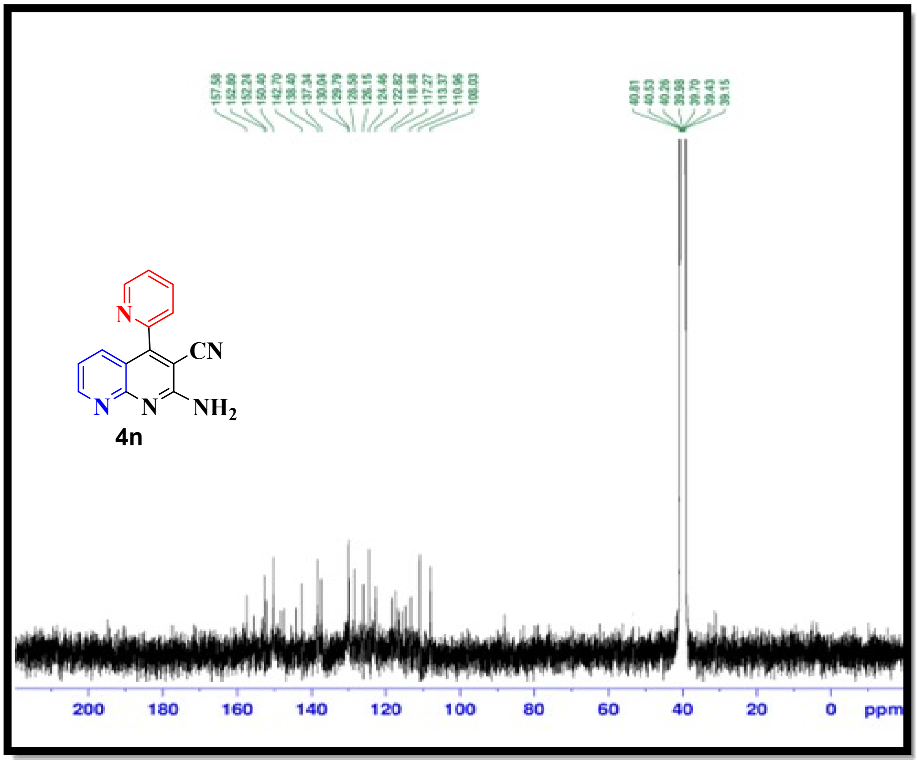
157.58
152.80
152.24
150.40
142.70
138.40
137.34
130.04
129.79
126.58
126.15
124.46
122.82
118.48
117.27
113.37
110.96
108.03
40.81
40.53
40.26
39.98
39.70
39.43
39.15
CN
NH2
4n
200
180
160
140
120
100
80
60
40
20
0
ppm
